# Injectable bottlebrush hydrogels with tissue-mimetic mechanical properties

**DOI:** 10.1126/sciadv.abm2469

**Published:** 2022-01-21

**Authors:** Foad Vashahi, Michael R. Martinez, Erfan Dashtimoghadam, Farahnaz Fahimipour, Andrew N. Keith, Egor A. Bersenev, Dimitri A. Ivanov, Ekaterina B. Zhulina, Pavel Popryadukhin, Krzysztof Matyjaszewski, Mohammad Vatankhah-Varnosfaderani, Sergei S. Sheiko

**Affiliations:** 1 Department of Chemistry, University of North Carolina at Chapel Hill, Chapel Hill, NC 27599-3290, USA.; 2 Department of Chemistry, Carnegie Mellon University, 4400 Fifth Avenue, Pittsburgh, PA 15213, USA.; 3 Phystech School of Electronics, Photonics, and Molecular Physics, Moscow Institute of Physics and Technology, Institutskiy per. 9, Dolgoprudny 141700, Russia.; 4 Institute of Problems of Chemical Physics, Russian Academy of Sciences, Chernogolovka 142432, Russia.; 5 Institut de Sciences des Matériaux de Mulhouse-IS2M, CNRS UMR 7361, 15 rue Jean Starcky, F-68057 Mulhouse, France.; 6 Faculty of Chemistry, Lomonosov Moscow State University, Leninskie Gory 1/51, Moscow 119991, Russia.; 7 Institute of Macromolecular Compounds, Russian Academy of Sciences, St. Petersburg 199004, Russia.

## Abstract

Injectable hydrogels are desired in many biomedical applications due to their minimally invasive deployment to the body and their ability to introduce drugs. However, current injectables suffer from mechanical mismatch with tissue, fragility, water expulsion, and high viscosity. To address these issues, we design brush-like macromolecules that concurrently provide softness, firmness, strength, fluidity, and swellability. The synthesized linear-bottlebrush-linear (LBL) copolymers facilitate improved injectability as the compact conformation of bottlebrush blocks results in low solution viscosity, while the thermoresponsive linear blocks permit prompt gelation at 37°C. The resulting hydrogels mimic the deformation response of supersoft tissues such as adipose and brain while withstanding deformations of 700% and precluding water expulsion upon gelation. Given their low cytotoxicity and mild inflammation in vivo, the developed materials will have vital implications for reconstructive surgery, tissue engineering, and drug delivery applications.

## Teaser

Minimally invasive injection yields robust hydrogels that mimic the mechanics and water fraction of surrounding tissue.

## INTRODUCTION

**Fig. 1.**
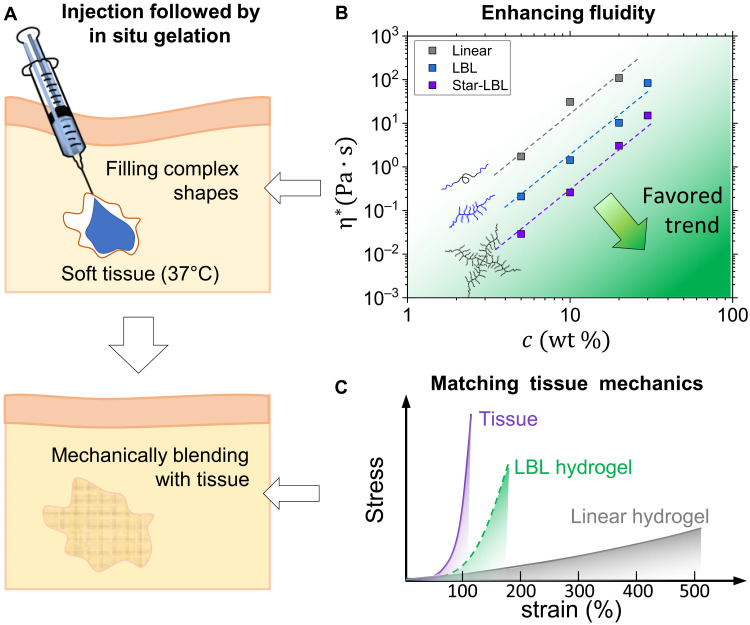
Injectable hydrogels. (**A**) Injection of a polymer solution at room temperature followed by in situ gelation in a target environment such as biological tissue at 37°C. The formed hydrogel has a well-defined interface with the surrounding tissue while matching its mechanical properties. (**B**) Complex viscosity as a function of solution concentration for three block copolymer architectures: linear PNIPAM-PEG-PNIPAM triblock (*M*_n_ = 65 kDa), LBL PNIPAM-bbPEG-PNIPAM triblock (*M*_n_ = 432 kDa), and four-arm star with bbPEG-PNIPAM diblock arms (*M*_n_ = 472 kDa) (figs. S1 to S4). The arrow indicates the favored trend in the injectable hydrogel design: achieving lower viscosity at a higher concentration to facilitate in vivo injections and three-dimensional (3D) printing applications. Despite higher molecular weight, brush polymer solutions are more fluid compared to their linear counterparts at the same concentration. (**C**) Mechanical mismatch between linear hydrogels and biological tissues. The LBL systems studied in this paper mimic both the initial softness and J-shape deformation response of soft tissues.

Injectable hydrogels are aqueous polymer solutions that undergo in situ gelation upon deployment to their target environment, such as the human body or porous scaffolds (Fig. 1A). These materials are vital for reconstructive surgery, tissue engineering, and drug delivery applications as they are minimally invasive, allow filling of irregular cavities, and enable co-injection of drugs and biologics (*1*–*7*). The successfully implemented gelation mechanisms carry distinct pros and cons (*4*, *5*, *8*–*10*). For example, covalent crosslinking yields mechanically robust gels, but the uncontrolled leaching of residual crosslinkers, initiators, and monomers is harmful to surrounding tissue (*11*). On the other hand, physically crosslinked gels are formed under innate conditions, and hence are much safer, but they suffer from mechanical weakness, which may lead to fragmentation and migration through the body (*12*, *13*). Both classes of injectable hydrogels, composed of linear polymers, require a substantial amount of water (90 to 99%) to lower the solution viscosity (Fig. 1B) and mimic the characteristic modulus (10 to 1000 Pa) of supersoft tissues such as adipose, brain, and lung tissue. This water content notably exceeds tissues’ 70%, which may lead to implant shrinkage due to water expulsion. Furthermore, synthetic hydrogels demonstrate a considerable mechanical mismatch with surrounding tissues (*14*, *15*), which is identified as one of the leading causes of inflammation in reconstructive surgery as well as inadequate cell response in tissue engineering (*16*–*19*). All soft tissues demonstrate the characteristic J-shape stress-strain curves, where the initially soft response (~1 to 10 kPa) is followed by intense strain stiffening, also known as firmness, whereby the modulus increases by two or three orders of magnitude (Fig. 1C) (*20*–*23*). In contrast, while various polymeric systems succeed in achieving soft hydrogels (*24*–*29*), combining this with tissue-mimetic firmness remains challenging for synthetic systems (*30*–*32*).

Here, we introduce a distinct injectable hydrogel platform based on hydrophilic thermosensitive linear-bottlebrush-linear (LBL) triblock copolymers that self-assemble at body temperature. Unlike current approaches to the design of soft tissue fillers, the developed systems combine vital advantages such as (i) the tissue-mimetic J-shape deformation response at a controlled water fraction, which reduces the mechanical mismatch between the implant and the native tissue; (ii) low viscosity at high polymer concentration to facilitate injectability; (iii) physical crosslinking that eliminates leaching of chemicals into the body; and (iv) mechanical resilience that allows withstanding up to 700% deformations at a low modulus below 1 kPa.

## RESULTS

The combination of linear and bottlebrush blocks is pivotal in providing multiple vital improvements to injectable hydrogels. First, brush macromolecules are more compact and less entangled (*33*–*37*), which facilitates injection by reducing solution viscosity compared to linear counterparts of the same molecular weight and concentration (Fig. 1B) (*38*). Second, grafted side chains, acting as crosslinker diluents, alleviate the need for a large water content to attain the desired gel softness (*14*). Third, steric repulsion between densely grafted side chains in the brush block instigates strain-stiffening of polymer networks at much lower strains of <200% (Fig. 1C) (*39*). Like softness, strain stiffening can also be adjusted in a broad range by varying the brush architecture (*15*). Fourth, both chemical and architectural dissimilarity of linear and bottlebrush blocks results in strong microphase separation (*40*, *41*), which sustain up to 700% deformation as discussed later. Fifth, the softness and disentangled nature of brush networks enhance their swellability, allowing equilibrium swelling ratios up to *V*_gel_/*V*_dry_ = 40 (*42*), which eliminates the after-gelation syneresis.

**Fig. 2.**
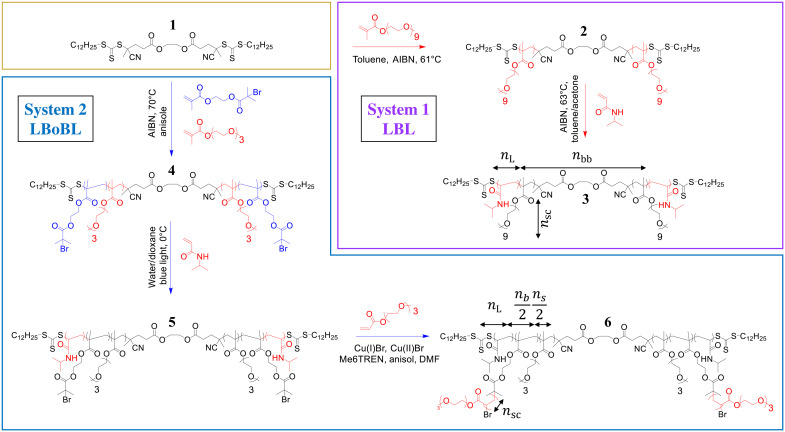
Synthesis of thermoresponsive copolymers. A bifunctional CTA (bfCTA) (**1**) is prepared via an esterification procedure (Materials and Methods). System 1: LBL triblock (LBL)—two-step RAFT polymerization of PEG methyl ether methacrylate (PEGMA; *M*_w_ = 500 g mol^−1^) yields bb-CTA (**2**) and PNIPAM linear blocks at both bottlebrush ends (**3**). System 2: LBoBL triblock (LBoBL)—three-step polymerization of PNIPAM-BoBPEG-PNIPAM triblock with ATRP functionality. bfCTA is used to synthesize comb-like middle block containing OEO_3_MA and branches with bromine functionality (**4**) required for polymerizing linear PNIPAM chains at both ends (**5**). ATRP polymerization of triethylene glycol methyl ether acrylate (PEGA; *M*_w_ = 218 g mol^−1^) (using Br-terminated side chains) allows the preparation of PNIPAM-BoBPEG-PNIPAM LBoBLs with *n*_sc_ = 13, 24, 67, and 126 (**6**). For more details, see Materials and Methods.

To demonstrate the effect of brush architecture on the gelation behavior and resulting mechanical properties, we synthesize two systems of hydrophilic triblock macromolecules with thermosensitive polyethylene glycol (PEG) bottlebrush block and poly(*n*-isopropylacrylamide) (PNIPAM) linear end blocks (Fig. 2). In system 1 (LBL), we use reversible addition fragmentation chain transfer (RAFT) to vary the degree of polymerization (DP) of the bottlebrush (bb) backbone, *n*_bb_= 274, 550, and 880, while maintaining the same DP of side chains at *n*_sc_ = 9 (Table 1). In system 2 [linear-brush-on-brush-linear (LBoBL)], we vary DP of side chains from *n*_sc_ = 13 to 126 at constant *n*_bb_ = 399 and *n*_L_ = 1200 by using a combination of atom transfer radical polymerization (ATRP) and RAFT, respectively. To prevent crystallization of longer PEG in system 2, we use the so-called brush-on-brush strategy, which yields comb-like polymers with brush-like side chains (*43*). In both systems, we deliberately keep the volume fraction of linear block (ϕ_L_) below 0.3 to ensure formation of spherical domains (*44*, *45*).

**Table 1.** Architectural parameters and gelation properties. Four series (A to D) of LBL and LBoBL triblocks with different degrees of polymerization (DP) of bottlebrush backbone (*n*_bb_), linear block (*n*_L_), and side chains (*n*_sc_), determined by NMR spectroscopy (figs. S5 and S6). In system 2 (series D), the brush block includes PEG side chains and PEG spacers between the side chains, resulting in *n*_bb_ = *n_b_* + *n_s_*, where indices *b* and *s* represent brush DP = 144 and spacer DP = 255, respectively. ϕ_L_ = *V*_lin_/(*V*_lin_ + *V*_bb_) is the volume fraction of linear blocks. *T*_gel_ is gelation temperature at the intersection of the *G*′(*T*) and *G*′′ (*T*) curves (Fig. 4, A and B). *G*_37_ corresponds to storage modulus measured at 37°C and 1 Hz. The NA (not applicable) cells in series A and C indicate that *T*_gel_ is outside the physiological range (*T*_gel_ > 37°C). See table S1 for the complete set of measurements at different concentrations.

**Series**	***n*_bb_**	***n*_sc_**	**ϕ_L_**	***n*_L_**	***T*_gel_ (°C)**	***G*_37_ (Pa)**	**Nomenclature**
System 1—at 20 wt %							
A	274	9	0.05	34	NA	NA	LBL-274-9-6%
			0.12	79	32.4	284	LBL-274-9-12%
			0.24	188	30.8	583	LBL-274-9-24%
B	550	9	0.05	60	33.2	119	LBL-550-9-6%
			0.12	169	32.4	317	LBL-550-9-12%
			0.20	310	29.6	713	LBL-550-9-24%
C	880	9	0.05	113	NA	NA	LBL-880-9-6%
			0.13	294	30.7	267	LBL-880-9-12%
			0.22	542	29.2	454	LBL-880-9-24%
System 2—at 10 wt %							
D	399	13	0.31	1200	33.6	1.7	LBoBL-399-13-31%
		24	0.22		35.2	3.7	LBoBL-399-24-22%
		67	0.10		32.9	141	LBoBL 399-67-10%
		126	0.06		33.4	147	LBoBL-399-126-6%

**Fig. 3.**
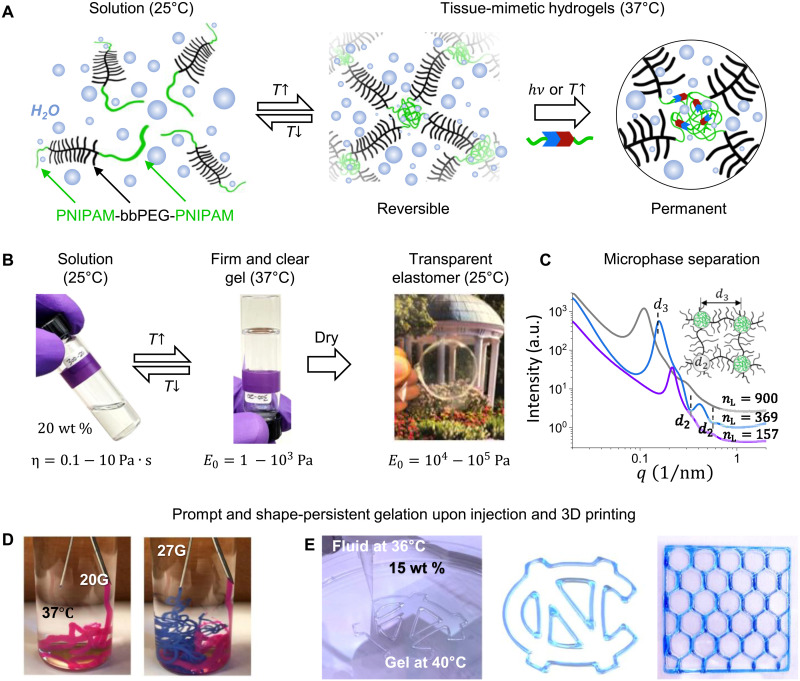
Tissue-mimetic thermosensitive hydrogels and elastomers. (**A**) LBL triblock copolymers with linear PNIPAM and bottlebrush PEG blocks form a homogeneous solution in water at 25°C. Upon heating above LCST, gelation occurs because of microphase separation of the PNIPAM blocks. LBL hydrogel implants can be either removed through cooling-induced network dissociation or permanently crosslinked by reactive monomeric units purposely incorporated within L-block. (**B**) Reversible transition between clear solutions of LBL triblocks at 25°C and transparent hydrogels at 37°C. After solvent evaporation, an optically transparent film is formed and placed over a picture of the University of North Carolina (UNC) well. Underneath the pictures, solution viscosity gel and Young’s modulus ranges of the synthesized LBLs are indicated (tables S1 and S2). (**C**) 1D SAXS curves of selected LBLs. The dashed lines mark the minima of the sphere form factor (*d*_2_) and the interference peak (*d*_3_) (*40*), related to the diameter and ordering of PNIPAM domains dispersed in the PEG bottlebrush matrix (inset), respectively. The PNIPAM domain size increases with *n*_L_ at an aggregation number of *Q* ≅ 150 (table S3), in agreement with the previously studied PMMA-bbPDMS-PMMA elastomers (*48*). a.u., arbitrary units. (**D**) Swift gelation of an LBL solution (20 wt %) upon injection into water at 37°C using two needle sizes, 20 gauge (20G) and 27 gauge (27G) (movie S1). Pink and blue organic dyes were added for optical distinction. (**E**) From left to right, 3D printing of the UNC logo with LBL hydrogel (27 gauge), the UNC logo printed with the same hydrogel mixed with the Brilliant Blue dye, and 30 mm × 30 mm × 1 mm honeycomb, respectively, using a Cellink Biox 3D printer.

Like other temperature-responsive copolymers (*44*, *46*, *47*), the PNIPAM-bbPEG-PNIPAM triblocks promptly self-assemble upon reaching their lower critical solution temperature (LCST) (Fig. 3A and fig. S7) to produce two types of polymer networks: hydrogels at body temperature and elastomers after water evaporation (Fig. 3B and movie S1). The gelation process is ascribed to LCST-triggered microphase separation of the PNIPAM linear (L) blocks, which results in a bottlebrush network, physically crosslinked by spherical L-domains corroborated by small-angle x-ray scattering (SAXS; Fig. 3C) and consistent with theoretical predictions (*40*, *41*, *48*). The proposed system benefits from PNIPAM’s distinct LCST transition within the physiological temperature range (*49*) yet readily allows application of other thermosensitive polymers such as poly(*N*-vinyl caprolactam) and polyoxazolines (*45*, *50*). Strong microphase separation upon temperature stimuli yields soft yet mechanically robust hydrogels that maintain their shape upon extrusion (Fig. 3, D and E, and movie S2). The optical transparency of both hydrogels and elastomers suggests the formation of homogeneous networks, in contrast to frequently observed turbidity upon microphase separation of linear counterparts (*51*–*53*).

**Fig. 4.**
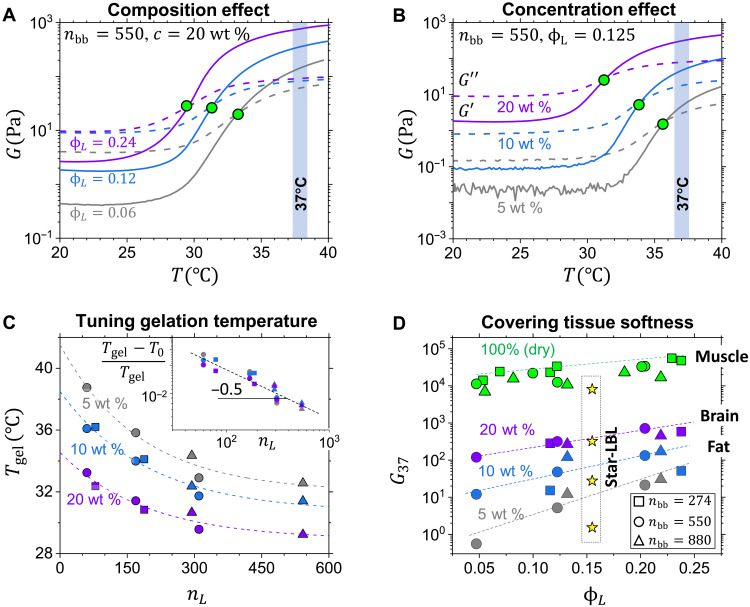
Gelation properties. Temperature sweeps of aqueous solutions of representative LBL copolymers (figs. S8 and S9) measured at (**A**) three different volume fractions of the linear PNIPAM block (LBL-550-9-*x*%) and (**B**) three different solution concentrations (LBL-550-9-12%), as indicated. Solid and dashed lines correspond to the storage and loss moduli (*G*′ and *G*′′), respectively. The vertical blue bars in (A) and (B) correspond to body temperature of 37°C. (**C**) The gelation temperature (*T*_gel_) decreases with DP of PNIPAM linear block (*n*_L_) at three different solution concentrations. The symbol shapes are defined in (D). Inset: Log-log plot of the relative gelation temperature τ = (*T*_gel_ − *T*_0_)/*T*_gel_ versus *n*_L_ demonstrates the square root decay as $\tau \sim n_{L}^{-0.5}$ (eqs. S1 to S7). (**D**) The gel storage modulus, *G*_37_, measured at 37°C, increases with both solution concentration and volume fraction of the PNIPAM block (ϕ_L_). The star symbols show *G*_37_ of hydrogels formed by self-assembly of four-arm block copolymers with bbPEG-PNIPAM diblock arms (*n*_bb_ = 687, *n*_L_ = 447, *n*_sc_ = 9, and ϕ_L_ = 0.22) (fig. S4).

The synthesized LBL triblocks were then dissolved in water at different concentrations to monitor the gelation process as a function of temperature, where the gelation temperature (*T*_gel_) is identified as intersection of the storage modulus, *G*′ (*T*), and loss modulus, *G*′′ (*T*), curves (Fig. 4, A and B, and figs. S8 to S10). The self-assembly process is reversible, as demonstrated both by returning to the initial modulus upon cooling and by prompt responses to cyclic temperature variations (fig. S11). Depending on LBL structural composition (Fig. 4A) or solution concentration (Fig. 4B), *T*_gel_ varies within a range of cloud points *T*_c_ ≅ 28 ° to 40 ° C (Fig. 4C, Table 1, and table S1). Like in all thermosensitive polymers (*54*, *55*), onset of gelation shifts to lower temperatures with increasing solution concentration (Fig. 4C). At a constant concentration, *T*_gel_ decreases with increasing linear block size, while the effects of *n*_bb_ and *n*_sc_ are marginal (Table 1, systems 1 and 2). The *n*_L_ effect is consistent with the theoretically predicted scaling relation $(T_{\text{gel}}-T_{0})/T_{\text{gel}}\sim n_{L}^{-1/2}$ (inset in Fig. 4C), where *T*_0_ corresponds to sign reversal of the second virial coefficient of NIPAM-NIPAM interaction (eq. S4 and fig. S18).

The gel storage modulus *G*_37_, measured at the physiological temperature of 37°C, shows strong dependence on both solution concentration and LBL composition (*n*_bb_, *n*_sc_, *n*_L_, and ϕ_L_), which allows covering the entire soft tissue range from 10 to 10^5^ Pa (Fig. 4D). Especially, strong effect is demonstrated by the lower concentration gels [5 weight % (wt %)] that cover two orders of magnitude in modulus upon increasing ϕ*_L_* from 0.05 to 0.25. The observed modulus increase is ascribed to the correspondingly increasing size of L-domains measured by SAXS (Fig. 3C and table S3), which corresponds to higher aggregation numbers, i.e., degree of crosslinking. Physical properties, such as solution viscosity and gel modulus, can be further adjusted using four-arm star-like LBL block copolymers (Figs. 1C and 4D, respectively), representing the scope of future studies.

**Fig. 5.**
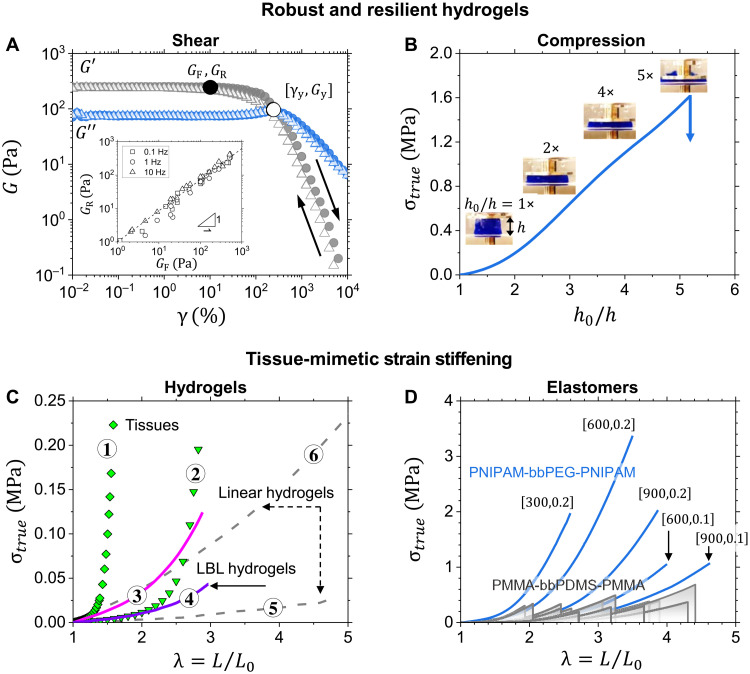
Mechanical properties. (**A**) Representative forward and reverse shear strain sweeps at 1 Hz give the yield strain (γ_y_) measured at the *G*′ and *G*′′ intersect (hollow circle) for PNIPAM-bbPEG-PNIPAM solution (*n*_bb_ = 550, ϕ_L_ = 0.12, *c* = 20 wt %, LBL-550-9-12%). Inset: Reverse (*G*_R_) versus forward (*G*_F_) taken at 10% strain at the same frequency (solid circle) calculated form and cycles (tables S4 to S6). This LBL hydrogel maintains linear viscoelasticity up to a shear strain of γ ≅ 100% (solid circle), followed by shear-thinning due to dissociation of the physical networks at γ ≅ 300% (hollow circle). (**B)** Uniaxial compression of a cylindrical sample of PNIPAM-bbPEG-PNIPAM (*n*_bb_ = 550, ϕ_L_ = 0.2, *c* = 15 wt %, LBL-550-9-24%) at 37°C in water (0.5 mm/s, *h*_0_ = 11.7 mm). Small amount of Brilliant Blue dye was added to the hydrogel for optical contrast. The corresponding true stress-compression curve along with the sample snapshots captured at different degrees of compression demonstrates material resilience up to 400% strain. (**C**) Stress-strain curves of chemically crosslinked LBL hydrogels (3 and 4) at *Q* = 3 versus linear hydrogels (5 and 6) (*58*, *59*) measured upon uniaxial extension. Curves 1 and 2 represent two biological tissues, 1 and 2, fetal membrane and gut, respectively (table S8). The linear hydrogels remain soft in a broad range of deformations, while the initially soft LBL stiffen with deformation, demonstrating the characteristic J-shape mechanical response. (**D**) Tensile stress-strain curves of PNIPAM-bbPEG-PNIPAM elastomers of different compositions [*n*_bb_,ϕ_L_] demonstrate much higher strength compared to the hydrophobic PMMA-bbPDMS-PMMA from (*40*).

The physically crosslinked gels are mechanically resilient—a vital feature for reconstructive body implants that are subjected to recurrent and large deformations—as confirmed by cyclic strain sweep tests showing a high yield strain of γ_y_ ≅ 300% (Fig. 5A). Depending on the LBL composition, the γ_y_ may reach up to 700% as observed for the sample, LBL-880-9-24% with *n*_bb_ = 880, ϕ_L_ = 0.22, and *G*_37_ = 450 Pa (tables S4 to S6 and figs. S12 and S13). The deformation-caused dissociation process is fully reversible, as demonstrated by *G*′ recovery upon strain decrease (Fig. 5A, inset; figs. S12, S13, and S23; and tables S4 to S6), indicative of prompt recovery upon dynamic deformation. The mechanical resilience is corroborated by uniaxial compression test, where an LBL hydrogel (15 wt %) withstands up to fourfold compression (Fig. 5B). This behavior is on par with covalently crosslinked hydrogels and represents a substantial improvement of physically crosslinked injectable hydrogels that are typically very brittle (*4*, *5*, *8*, *9*, *44*). Furthermore, the 10 to 30 wt % LBL hydrogels do not exhibit any sign of syneresis, which is attributed to a relatively high equilibrium swelling ratio ranging from 12 to 24 (table S7). This behavior is consistent with the swellability enhancement observed in chemical brush networks caused by disentanglement of bottlebrush networks strands and soft elasticity of brush networks (*42*).

Characterization of the strain-stiffening behavior requires tensile tests that are straightforward for dry elastomer but quite challenging for physically crosslinked LBL hydrogels due to their prompt dissociation upon removal from a thermal bath. To resolve this issue, we develop two complementary synthetic strategies for post-gelation crosslinking that, depending on application, enable either slow spontaneous or rapid on-demand fixation of LBL hydrogels. The first approach is based on the Diels-Alder (DA) chemistry involving the synthesis of two complementary LBL structures with furan and maleimide functional groups in their L-blocks that slowly (~10 hours) couple following the microphase separation above LCST (figs. S14 and S15). The second strategy is empowered by incorporating a controlled fraction of ultraviolet (UV)–curable methacrylate groups within the PNIPAM L-blocks that react on demand under mild UV irradiation (λ = 365 nm) (Materials and Methods; fig. S16). The formation of permanent networks is verified by swelling the crosslinked samples in organic solvents over a period of 7 days (fig. S17) without the loss of optical transparency (figs. S17 and S21). This allows tensile tests of LBL hydrogels and quantitative comparison of their deformation behavior with that of linear chain hydrogels and soft tissues (Fig. 5C and fig. S18). The LBL hydrogels demonstrate much steeper strain stiffening characterized by a firmness parameter of β ≅ 0.2, which is considerably higher than β ≅ 0.01 of linear hydrogels (table S8). The beta parameter depends on the microphase separated morphology (domain size, aggregation number), the interaction parameter, and the block dimensions. It can be enhanced by increasing the side-chain length and volume fraction of the linear blocks or by synthesizing brush blocks with shorter backbones (*36*). Although the superior firmness of tissues (β ≅ 0.4 to 0.9) is not achieved, the current LBL hydrogels allow mimicking tissues such as lung and gut that are located at the lower boundary of the tissue firmness range (*14*).

After water evaporation, we obtain dry transparent elastomers (Fig. 5D) that are stiffer and more strain stiffening than the corresponding LBL hydrogels (table S2). Like the hydrogels, they exhibit the characteristic J-shape stress-strain response matching that of stiffer tissues such as porcine skin and aorta (fig. S19). Furthermore, the PNIPAM-bbPEG-PNIPAM elastomers are significantly stronger than their PMMA (polymethyl methacrylate)–bbPDMS (polydimethylsiloxane)–PMMA counterparts (*40*) as they attain a stress at break of 3.5 MPa, notably higher than the 0.6 MPa of the PMMA/PDMS systems. The strength enhancement is ascribed to (i) hydrogen bonding between PNIPAM chains and brush PEGs upon progressive withdrawal of the PNIPAM linear block from L-domains upon deformation and (ii) well-defined structure of LBL due to a higher retention of chain-end functionality during the RAFT polymerization of bottlebrush backbone.

**Fig. 6.**
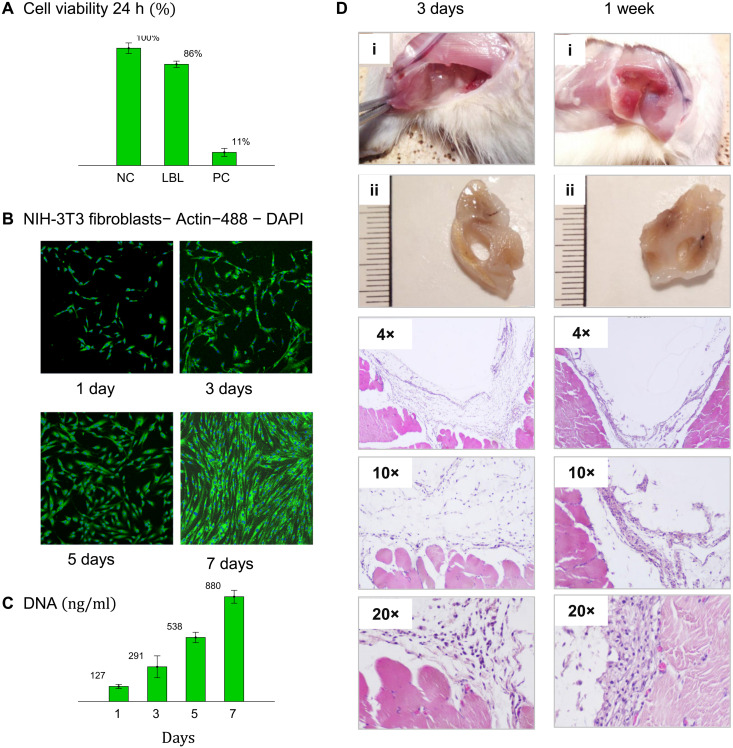
Toxicity and in vivo assessment. (**A**) Cytotoxicity assay shows 86% viability of NIH/3T3 fibroblasts exposed to 24-hour aqueous extracts from a PNIPAM-bbPEG-PNIPAM hydrogel (*n*_bb_ = 336, *n*_L_ = 502, ϕ_L_ = 0.26, *T*_gel_ = 32 ° C, *E*_0, gel_ ~ 3 kPa, *E*_0, dry_ = 37.7 kPa, *G* = 8.4 kPa, β = 0.25) relative to negative and positive counts (NC and PC). (**B**) Proliferation of the cells cultured on a PNIPAM-bbPEG-PNIPAM sample is monitored by fluorescent microscopy over a period of 7 days (Materials and Methods). (**C**) DNA concentration in the cultured cells increases over a period of 7 days, which corroborates the cell proliferation. (**D**) Tissue examination 3 days and 1 week after intramuscular injection of LBL hydrogel (*n*_bb_ = 587, *n*_L_ = 461, ϕ_L_ = 0.26, *T*_gel_ = 32 ° C, *E*_0, gel_ ~ 2 kPa, *E*_0, dry_ = 106 kPa, *G* = 20.3 kPa, β = 0.32), which solidifies at body temperature and returns to the liquid state upon explantation. Clinical examination of (i) surgical site and (ii) explanted specimens suggests that the intramuscular implants are localized within a confined injection cavity. The micrographs of different magnifications (as indicated) show tissue cross-sections stained with hematoxylin and eosin. At lower magnifications, the injected LBL hydrogels are seen to form a well-defined oval-shaped cavity surrounded by a moderate capsule. The histology evaluation showed the slight to moderate inflammatory response with a tendency to decline.

Biocompatibility of the LBL hydrogels is validated by cytotoxicity tests (ISO 10993-5) and in vivo injection to animal models (Fig. 6). NIH/3T3 fibroblasts demonstrate 86% viability when exposed to aqueous extracts from an LBL hydrogel for 24 hours (Fig. 6A). Cells’ proliferation on LBL substrates is monitored by fluorescence microscopy over the course of 7 days (Fig. 6B) and further corroborated by the progressively increasing DNA content in cultured cells over the time of experiment (Fig. 6C). To evaluate the tissue reaction to LBL hydrogels, we examine animal models subjected to intramuscular injection followed by prompt gelation at body temperature. Figure 6D shows explanted samples and the micrographs of tissue cross-sections taken at ×4, ×10, and ×20 magnifications harvested after 3-day and 1-week implantation, inflammatory phase of the wound healing process (*56*). Upon clinical examination, the samples are localized at the injected site, forming a round cavity without visible spreading into the surrounding tissue. After 1 week, the samples are found to be fully intact and distinctly separated from surrounding muscle tissue. The injected samples are well tolerated, with no evidence of clinically visible inflammatory response or necrosis in surrounding tissues (Fig. 6D, i and ii). According to the histological analysis of the specimens stained with hematoxylin and eosin (H&E), injected hydrogels are intact and surrounded by a smooth capsule at 3 days and 1 week after surgical injection. The capsule presents a layer of young connective tissue with a small amount of loose subtle collagen fibers, infiltrated with macrophages and a small number of lymphocytes. No multinucleated foreign body giant cells are observed. After 1 week, a tendency of declining inflammation is observed.

## DISCUSSION

Hydrophilic thermoresponsive LBL triblock copolymers afford a powerful platform for the design of injectable tissue-mimetic hydrogels and elastomers. Both the mechanical properties and gelation temperature can be finely tuned by the triblock architecture at a given solution concentration. Concentrated yet low viscosity aqueous solutions of LBL triblocks promptly respond to physiological temperature enabling injection along with prompt gelation at a controlled water fraction. Upon drying, thermoplastic elastomers are obtained, which can be either dissolved or molded at an elevated temperature. Both hydrogels and elastomers demonstrate tissue-like mechanical properties characterized by the oxymoronic combination of softness and firmness, resulting in the characteristic J-shape of stress-strain curves. Because of a high aggregation number *f* ~ 150 and the equilibrium nature of the gelation process, LBL hydrogels are mechanically robust, sustaining ~700% deformation and promptly reassociating if ruptured. By incorporating UV- or temperature-triggered crosslinkers in L-domains, reversible networks can be converted to permanent hydrogels that resist accidental temperature variations. As supported by in vitro and in vivo studies, the injectable hydrogels can be used as either body fillers for reconstructive surgery or bio-inks when mixed with cells for tissue engineering. These materials are readily adaptable for additive manufacturing of shape-specific objects such as organs.

## MATERIALS AND METHODS

### Materials

PEG methyl ether methacrylate [PEGMA; *M*_w_ = 500 g mol^−1^; 200 parts per million (ppm) hydroquinone monomethyl ether (MEHQ) as inhibitor] was purchased (Sigma-Aldrich) and purified passing through an aluminum oxide column (twice). NIPAM (97%, *M*_w_ = 113.16 g mol^−1^) was purchased from Sigma-Aldrich and TCI America and recrystallized using a 50:50 mixture of toluene/hexane (three times) for purification. Potassium *tert*-butoxide (reagent grade, ≥98%; Sigma-Aldrich) iodine (flakes, ReagentPlus, ≥99%; Sigma-Aldrich) and 2,2′-azobis(2-methylpropionitrile) (AIBN; 98%; Sigma-Aldrich) as the RAFT initiator were used as received. For UV chemical crosslinking, UV photocrosslinker Irgacure 2595 (BASF) was used. Triethylene glycol methyl ether methacrylate (OEO_3_MA; 93%; Sigma-Aldrich) and triethylene glycol methyl ether acrylate (OEO_3_A; 95%; TCI) were passed through basic alumina plugs to remove inhibitor. Copper(I) bromide (CuBr; 99.999%; Sigma-Aldrich), copper(II) bromide (CuBr_2_; 98%; Acros), α-bromoisobutyryl bromide (98%; Sigma-Aldrich), ethylene glycol (99.8%; Sigma-Aldrich), tris[2-(dimethylamino)ethyl]amine (Me_6_TREN; Sigma-Aldrich), *N*,*N*′-dicyclohexylcarbodiimide (DCC), 4-(dimethylamino)pyridine (DMAP; 98%; Sigma-Aldrich), 2-hydroxyethyl methacrylate (HEMA; >99%; Sigma-Aldrich), ethylene diamine (for synthesis; Sigma-Aldrich), anhydrous ethylene glycol (Sigma-Aldrich; 99.8%), ethanol amine (Sigma-Aldrich, ≥98%), furan (Acros, 99%; Sigma-Aldrich), maleic anhydride (99%; Sigma-Aldrich), isophorone diisocyanate (TCI, 98%; Sigma-Aldrich), furfuryl isocyanate (97%; Sigma-Aldrich), 2-isocyanatoethyl methacrylate (≤0.1% 2(3)-t-butylhydroquinone monomethyl ether (BHT) as inhibitor, 98%; Sigma-Aldrich), 4,4′-azobis(4-cyanovaleric acid) (≥98%; Sigma-Aldrich), dibutyltin dilaurate (DBTDL; TCI, 95%; Sigma-Aldrich), and PEG bis[2-(dodecylthiocarbonothioylthio)-2-methylpropionate] (average *M*_n_ = 10,800) were used as received without further purification. Reagent-grade toluene, anisole, tetrahydrofuran (THF), hexane, ethyl acetate, heptane, carbon disulfide, dichloromethane (DCM), methanol, and acetone were used as organic solvents for polymerization and purification purposes.

### Synthesis

#### Chain transfer agent

In a typical synthesis, 1-dodecanethiol (10.5 ml, 0.044 mol) is added over 5 to 10 min to a cold mixture of potassium *tert*-butoxide (4.932 g, 0.044 mol) and 200 ml of heptane in a 250-ml flask, under constant stirring. After 1 hour, within a period of 10 min, CS_2_ (3.3 g, 0.044 mol) is added and stirred for 30 min and then at room temperature for an additional 1 hour. To the slurry, iodine (6 g, 0.024 mol) is added and stirred overnight. The mixture was washed with deionized water (three times) and once with saturated water sodium thiosulfate. To the dried product (5 g, 9 mmol) in 100 ml of ethyl acetate, 4,4′-azobis(4-cyanovaleric acid) (9.9 mmol, 2.8 g) is added and refluxed at 88°C overnight. The product was washed with water (three times) and then dried with magnesium sulfate. The purified chain transfer agent (CTA) was recrystallized in hexane.

#### Bifunctional CTA

In a 100-ml round-bottom flask equipped with magnet and stopper, ethylene glycol (1.78 mmol, 0.111 g), DCC (3.72 mmol, 0.767 g) is added with 30 ml of dry DCM as solvent. Diluted CTA (3.72 mmol, 1.5 g) in 10 ml of DCM is added dropwise in a period of 10 min followed by one-shot injection of catalytic amount of DMAP (0.37 mmol, 0.045 g) diluted in 10 ml of DCM. The reaction was stirred overnight at room temperature. Afterward, the organic layer is washed with water (three times) and further purified using column chromatography (50:50 ethyl acetate/hexane).

#### Tetrafunctional star-CTA

In a 100-ml round-bottom flask equipped with magnet and stopper, pentaerythritol (0.91 mmol, 0.124 g), DCC (3.72 mmol, 0.767 g) is added with dry pyridine as solvent. CTA (3.72 mmol, 1.5 g) diluted in 10 ml of DCM was added dropwise in a 10-min period followed by one-shot injection of DMAP (0.37 mmol, 45 mg) diluted in 10 ml of DCM. Reaction proceeds overnight; afterward, organic layer is washed with water (three times) and further purified using column chromatography (50:50 ethyl acetate/hexane).

#### Linear PNIPAM-PEG-PNIPAM

In a typical synthesis, in a Schlenk flask equipped with stir bar, 1 g (92.6 μmol) of PEG bis[2-(dodecylthiocarbonothioylthio)-2-methylpropionate] is dissolved in 6 ml of 1,4-dioxane with 1 g (8.83 mmol) of NIPAM followed by 9.26 μmol (1.5 g) of AIBN. The mixture is purged with nitrogen for 1 hour, and the reaction started by lowering the flask in 65°C oil bath. The reaction stopped at desired conversion [checked with ^1^H nuclear magnetic resonance (NMR) spectroscopy; fig. S1] and precipitated in hexane three times.

#### LBL triblocks (system 1)

Step 1: In a typical synthesis, a 100-ml Schlenk flask was equipped with a stir bar, and a solution of monomer (PEG methacrylate methyl ether, 0.02 mol), bifunctional CTA (bfCTA;0.05 mmol), initiator (AIBN, 0.005 mmol), and toluene (3:1 monomer) was added and sparged with nitrogen for 1 hour. Polymerization was started by placing the flask in an oil bath at 61°C. Polymerization was stopped by exposing to air at 75% conversion (tracked using ^1^H NMR). Purified polymer was precipitated from toluene/hexane (three times) yielding bb-CTA. Step 2: In a typical synthesis, a 100-ml Schlenk flask was equipped with a stir bar, bb-CTA (0.005 mol), and NIPAM (0.0052 mol). The solids were dissolved in a solution of toluene/acetone/PEG (3:1:1) with AIBN (0.0005 mol) as initiator. The solution was sparged with nitrogen for 1 hour. Polymerization started by placing the flask in an oil bath at 63°C. After 3 hours, the polymer was precipitated in acetone/hexane three times. The resulting polymer is a triblock copolymer, PNIPAM-bbPEG-PNIPAM, dried by casting into films using Teflon petri dish. The volume fraction of the L-block (ϕ_L_) was determined via ^1^H NMR (fig. S5).

#### LBoBL triblocks (system 2)

Step 1: a 100-ml Schlenk-modified flask was loaded with OEO_3_MA (40.9 ml, 0.2 mol), 2-(2-bromoisobutryloxy)ethyl methacrylate (13.7 ml, 64 mmol), AIBN (2.5 mg, 15 μmol), bfCTA [2 ml of a stock solution (0.111 g ml^−1^) in *N*,*N*′-dimethylformamide (DMF), 0.26 mmol], anisole (50 ml), and a magnetic stir bar. The mixture was degassed by three cycles of freeze-pump thaw. The reaction was started by immersion in an oil bath set to 70°C. Polymerization was stopped once conversion reached 39.9% by ^1^H NMR spectroscopy. The crude product was purified by dialysis against a 1-kDa MWCO RC-treated dialysis membrane in a 50% methanol/THF solution. The product was dried overnight to yield the P(BiBEM_144_-co-OEO_3_MA_255_) backbone as a viscous oil. Purity was confirmed by ^1^H NMR. Step 2: A 1000-ml Schlenk-modified flask was loaded with NIPAM (133.78 g, 1.18 mol) and a magnetic stir bar. The NIPAM was dissolved in 530 ml of deionized water and cooled in an ice bath before P(BiBEM_144_-co-OEO_3_MA_255_) (5.6 g, 116 μmol of trithiocarbonate chain ends assuming the polymer has a *M*_n, th_ = 96,850) was added as a stock solution in dioxane (80 ml) with an additional 20 ml of DMF. The flask was wrapped in aluminum foil and sparged with nitrogen gas for 4 hours in a large ice bath. The flask and ice bath were loaded into a blue light chamber. The reaction was started by turning on the blue light (λ_max_ = 460 nm, intensity = 14.9 mW/cm^2^) and stopped after 35 min. The crude product was purified by dialysis against a 2-kDa RC-treated dialysis membrane in a 70% methanol/THF solution with six solvent changes and precipitated into diethyl ether three times. Purity was confirmed by ^1^H NMR. Step 3: In a typical synthesis, PNIPAM_1200_-*b*-P(BiBEM_144_-*co*-OEO_3_MA_255_)-*b*-PNIPAM_1200_ (1.98 g, 0.79 mmol, BiBEM) and copper(II) bromide (22 mg, 99 μmol) were added to a 100-ml Schlenk flask with a magnetic stir bar. The solids were dissolved in DMF (12 ml), anisole (50 ml), OEO_3_A (16.7 ml, 79 mmol), and Me_6_TREN (82 μl, 0.3 mmol). The flask was sealed and degassed by freezing in liquid nitrogen, evacuation under vacuum, and thawing for a total of three rounds. On the last round, the flask was opened under a strong flow of nitrogen gas, and copper(I) bromide (28 mg, 0.2 mmol) was quickly added. Polymerization started at room temperature and was stopped once conversion of the side chain was of the desired length, by extrapolation to conversion by ^1^H NMR. The reaction mixture was quenched with air, diluted with THF, purified by dialysis against a 50-kDa RC-treated dialysis membrane in a 70% methanol/THF solution with six solvent changes, and precipitated into diethyl ether (figs. S6 and S22 and table S9). The recipe used to prepare the PNIPAM_1200_-*b*-P(BiBEM-*g*-OEO_3_A_126_)_144_-*co*-OEO_3_MA_255_)-*b*-PNIPAM_1200_ brush used a higher molar ratio of [OEO_3_A]/[BiBEM] = 200.

#### Star-like LBL

The procedure follows synthesis of system 1 but with star-CTA as RAFT agent for synthesis of four-arm backbone, followed by using four-arm bottlebrush-CTA as macroinitiator to grow linear PNIPAM domains (fig. S4).

#### Isocyanate-terminated maleimide adduct for DA crosslinking (system 1)

In a typical synthesis, maleic anhydride (10 g, 0.102 mol) is initially protected with furan (21 g, 0.306 mol) in 100 ml of toluene in a 250-ml round-bottom flask at 80°C for 24 hours. Afterward, the solvent is fully evaporated, and the product is recrystallized twice from cold acetone (3,6-exo-1,2,3,6-tetrahydrophthalic anhydride). The product (10 g, 0.06 mol) is refluxed in 100 ml of methanol with dropwise addition of ethanol amine (0.12 mol, 7.35 g) in ice bath for 30 min at room temperature and refluxed at 80°C for 24 hours. Afterward, the solvent is fully evaporated, and the product is recrystallized from ethanol and chloroform twice forming protected 2-hydroxyethyl-maleimide (PHEMI). In 50 ml of dry DCM, PHEMI (10 g, 0.048 mol) is dissolved with catalytic amount of dibutyltin dilaurate. Excess isophorone diisocyanate (0.48 mol, 106 g) diluted in 10 ml of dry DCM is added dropwise over 30 min. Reaction is left at room temperature for 24 hours. Afterward, the solvent is evaporated using nitrogen and the product is washed with minimal amount of DCM and excess dry hexane (fig. S15).

#### DA crosslinking of tissue-mimetic hydrogel

The LBL triblock is functionalized with copolymerization of HEMA (compound 7, <5 mol %; fig. S8) during the second step of RAFT (system 1) and is modified via postpolymerization. The functionalized LBL is purged with nitrogen and reacted with excess AIBN at 65°C overnight to remove CTA end groups and cap both chain ends. The product is treated with excess maleimide-isocyanate and furfuryl isocyanate DA adducts in two separate reactions with DCM as the solvent at room temperature and catalytic amount of DBTDL. LBLs participated with hexane, and the two polymers with DA moieties were mixed and cast at room temperature to afford linear domain crosslinked bottlebrush elastomer (fig. S14).

#### UV chemical crosslinking of tissue-mimetic hydrogel

In a typical procedure, OH-functionalized capped LBL form system 1 is dissolved in deionized water, mixed with a water-soluble photoinitiator (Irgacure 2595, BASF), casted in a Teflon petri dish, and placed in an oven at 37°C for 1 hour to ensure macrophase separation. The physically crosslinked gel was quickly placed under UV for 2 min under nitrogen. The resultant gel is chemically crosslinked in linear domains (fig. S16).

### Dynamic mechanical analysis

Samples were cast from THF with concentration of ~20% and dried overnight on a balanced table. Dumbbell shapes with dimension of 12 mm × 2 mm × 1 mm were cut from the film for mechanical analysis. Samples were uniaxially extended up to the breaking point using an RSA-G2 dynamic mechanical analysis (DMA) system from TA Instruments at a constant strain rate of 0.005 s^−1^ and ambient temperature of 20° to 21°C. All samples were measured at least three times (except one sample with two measurements). To measure stress strain of LBL hydrogels at room temperature, chemically crosslinked dog bones were initially cut out of dry elastomer, swollen to *Q* = 3, and subjected to uniaxial tensile test. To measure compressive stress strain of physically crosslinked hydrogel, a 6-ml syringe containing the 15 wt % LBL solution was placed in an oven at 40°C for 10 min. The luer lock was cut, and the gel was plunged into heated medium (water at 40°C). The container was placed on DMA module and subjected to compressive strain.

### Rheology

To study the rheological behavior of the hydrogels, three different samples with concentrations of 5, 10, and 20% in water for each of the synthesized polymers were prepared. All measurements were done using AR-G2 rheometer (TA Instruments) equipped with stainless-steel plate (40 mm diameter) equipped with a solvent trap to prevent excess evaporation in higher temperatures. We measure storage and loss modulus (*G*′ and *G*′′, respectively) for a range of temperature and frequencies. Temperature measurements ranging from 20° to 40°C with a rate of 1°C/min at a frequency of 1 Hz were carried out. The dependency of storage and loss modulus on the strain-frequency variation was done for three representative frequencies of 0.1, 1.0, and 10 Hz at a constant temperature of 37°C. For this study, the applied strain was varied from 0.01 to 10,000%. In addition, the response of the synthesized hydrogels was assessed with time-temperature sweep. In this regard, initial temperature was set to 20°C and ramped up to 37°C while sampling over time.

### X-ray scattering measurements and analysis

The small-angle x-ray (SAXS) measurements were carried out at the ID02 beamline of the European Synchrotron Radiation Facility in Grenoble, France. The experiments were conducted in transmission geometry using a photon energy of 12.4 keV. The recorded 2D data were centered, calibrated, regrouped, and reduced to 1D using the SAXS utilities platform described elsewhere (*57*). The analysis of the SAXS data was performed using the small-angle neutron scattering (SANS) and Ultra-Small-Angle Neutron Scattering (USANS) data reduction and analysis package provided by NIST for the Igor Pro environment (WaveMetrics Ltd.). The monochromatic x-ray beam was collimated on the sample to a footprint of 50 μm × 100 μm. Hybrid photon-counting detector Eiger2 4M (Dectris Ltd., Switzerland) was implemented in the vacuum flight tube at a 3-m sample-detector distance. Because of high flux, the acquisition times were less than 10 ms. No specific radiation damage was observed during the measurements. For quantitative analysis of SAXS curves, we model the scattering intensity as *I*(*q*) ≈ Φ(*q*)*S*(*q*), where *S*(*q*) is the structure factor and Φ(*q*) is the form factor of homogeneous monodisperse spheres. The polydispersity effect was incorporated by a convolution of the scattering intensity with the Gaussian size distribution functions.

### PrestoBlue assay

The cytotoxicity of extracts from the thermoresponsive gel was studied according to ISO standard 10993-5 to resemble the first 24 hours of cellular response of the clinical administration. The thermoresponsive gel samples were placed into the extraction medium containing Dulbecco’s modified Eagle’s medium (DMEM), supplemented with 10% fetal calf serum (FCS) and 1% mixture of penicillin/streptomycin (Sigma-Aldrich) at a concentration of 3 cm^2^/ml. The samples were stored at 37°C and 5% CO_2_ for 24 hours. The fibroblasts (NIH/3T3, American Type Culture Collection) were seeded on a 96-well plate at an initial concentration of 10^4^ cells/cm^2^ and incubated for 24 hours. Then, the culture media were replaced by the extract’s media and incubated at 37°C and 5% CO_2_. The cellular viability was analyzed after 24 hours by using PrestoBlue cell viability reagent (Invitrogen) according to the manufacturer’s protocol. At the end of incubation time, the culture medium was replaced with 10% of resazurin-based PrestoBlue reagent, and the fluorescence intensity was recorded with a microplate reader (BioTek Instruments) at 544-nm/590-nm excitation/emission wavelength after 30 min of incubation.

### DNA quantification assay

NIH/3T3 proliferation in contact with thermoresponsive gel was evaluated for 7 days. To analyze the cellular proliferation, NIH/3T3 fibroblasts were cultured at the density of 5 × 10^5^ cells/ml with DMEM basal culture medium supplemented with 10% FCS and 1% penicillin/streptomycin. The NIH/3T3 fibroblasts were incubated at 37°C and 5% CO_2_. To assess the cellular proliferation, the fibroblasts were collected at 3, 5, and 7 days for DNA quantification. The DNA content of the cells was measured using the Quanti-iT PicoGreen dsDNA Kit (Invitrogen) based on the manufacturer’s protocol. Further, immunohistochemical staining was performed to monitor the cell number using a Cytopainter Green Fluorescence F-actin staining kit and 4′,6-diamidino-2-phenylindole (DAPI) following the manufacturer’s instructions.

### In vivo study

For this study, male Wistar white rats (weight, 350 to 400 g; age, 6 to 7 weeks old) were used in accordance with principles of the European Convention, Strasbourg, 1986 and the Helsinki Declaration of the World Medical Association for the Humane Treatment of Animals 1996. The rats were kept in a humidity- and temperature-controlled environment, on a 12-hour/12-hour light/dark cycle, with food and water made available ad libitum. Under general anesthesia, the injectable thermoresponsive gel samples were injected in the musculus adductor magnus on both hindlimbs. After surgical administration, animals were kept in individual cages. The tissue surrounding injected thermoresponsive specimens were explanted at 3 days and 1 week after surgery. For histological tissue evaluation, the harvested tissue specimens were fixed in 10% neutral formalin (pH 7.4) and then dehydrated in a series of ascending ethanol solutions (50, 70, 90, and 100% ethanol, 5 min each). Then, the dehydrated specimens were embedded in paraffin. The 5-μm paraffin slides were sectioned and deparaffinized. The slides were then stained with H&E after rehydration in a graded ethanol solution series (100, 90, and 70% ethanol, 5 min each, dH_2_O for 10 min). Tissue sections were studied using light microscopy (Leica), and micrographs of tissue cross-sections were taken at ×4, ×10, ×20, and ×40 magnifications from each time point. Institutional animal care and use committee guidelines were followed with animal subjects.

## Supplementary Materials

### Other Supplementary Material for this manuscript includes the following:

MDAR Reproducibility Checklist
