# Supplementary material for: Injectable bottlebrush hydrogels with tissue-mimetic mechanical properties
Source: Sci Adv. 2022 Jan 21;8(3):eabm2469. doi: 10.1126/sciadv.abm2469 (PMC8782458; doi:10.1126/sciadv.abm2469)
Supplement: Supplementary file 1 — Supplementary Text Figs. S1 to S23 Tables S1 to S9 Legends for movies S1 and S2 [file sciadv.abm2469_sm.pdf]

Science Advances
AAAS

Supplementary Materials for

Injectable bottlebrush hydrogels with tissue-mimetic mechanical properties

Foad Vashahi, Michael R. Martinez, Erfan Dashtimoghadam, Farahnaz Fahimipour, Andrew N. Keith, Egor A. Bersenev, Dimitri A. Ivanov, Ekaterina B. Zhulina, Pavel Popryadukhin, Krzysztof Matyjaszewski*, Mohammad Vatankhah-Varnosfaderani*, Sergei S. Sheiko*

*Corresponding author. Email: sergei@email.unc.edu (S.S.S.); mvatan@live.unc.edu (M.V.-V.); matyjaszewski@cmu.edu (K.M.)

Published 21 January 2022, *Sci. Adv.* **8**, eabm2469 (2022)
DOI: 10.1126/sciadv.abm2469

The PDF file includes:

Supplementary Text
Figs. S1 to S23
Tables S1 to S9
Legends for movies S1 and S2

Other Supplementary Material for this manuscript includes the following:

Movies S1 and S2

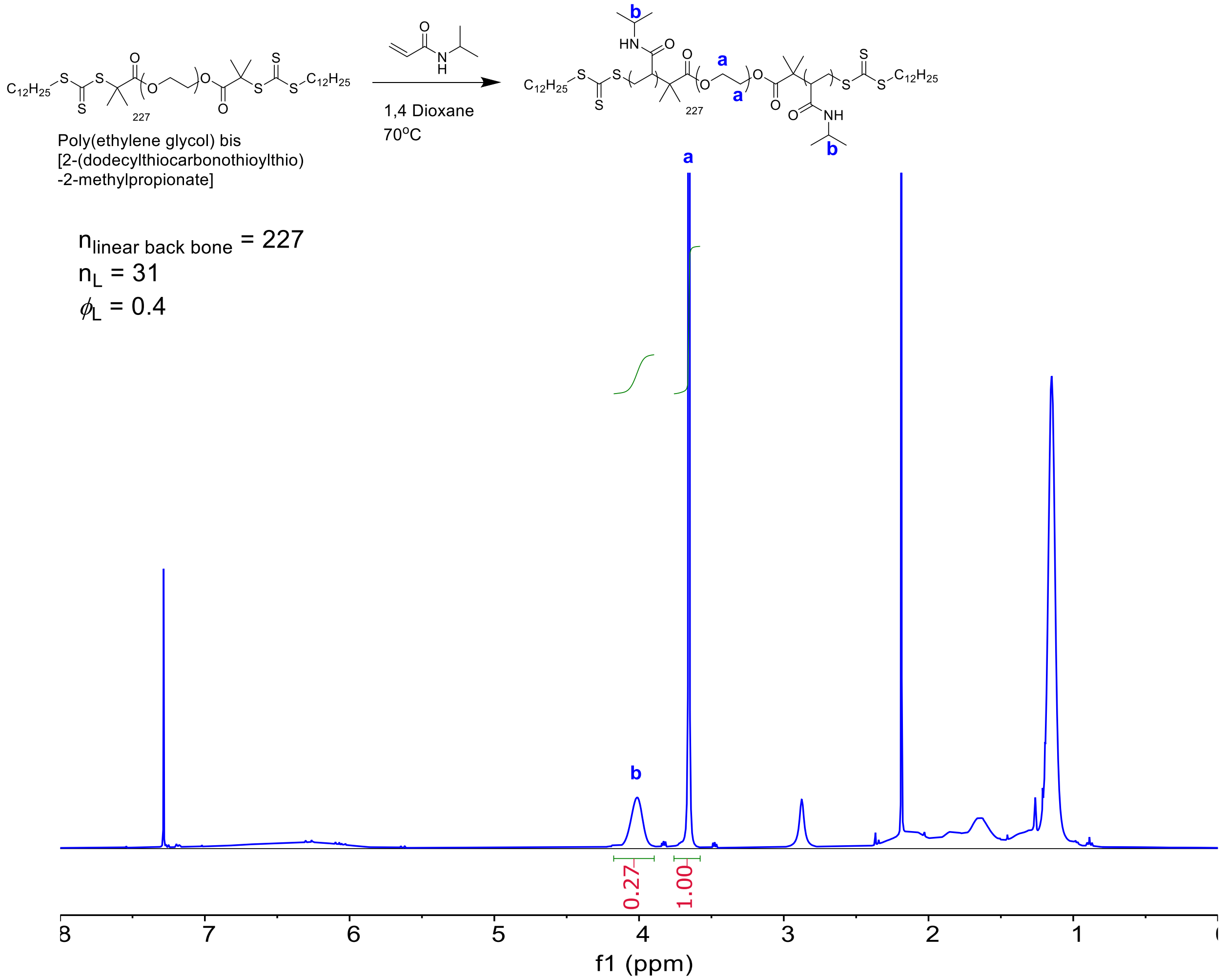

Fig. S1.

NMR spectra of linear PNIPAM-PEG-PNIPAM ABA triblock. $DP_{center\ block} \sim 227$, $n_L = 31$, $\phi_L \sim 0.4$ for different concentrations.

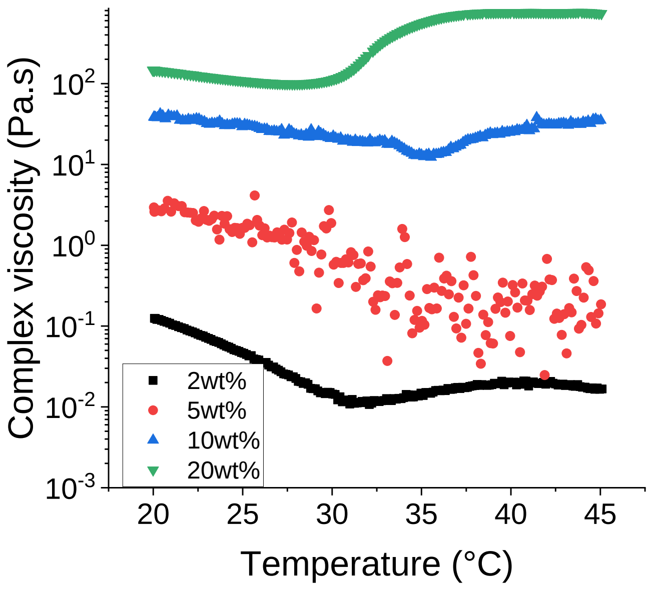

Fig. S2.

Complex viscosity of Linear ABA at different concentrations. Complex viscosity extracted from temperature sweep of linear PNIPAM-PEG-PNIPAM triblock, $DP_{center\ block} \sim 227$, $n_L = 31$, $\phi_L \sim 0.4$ for different concentrations.

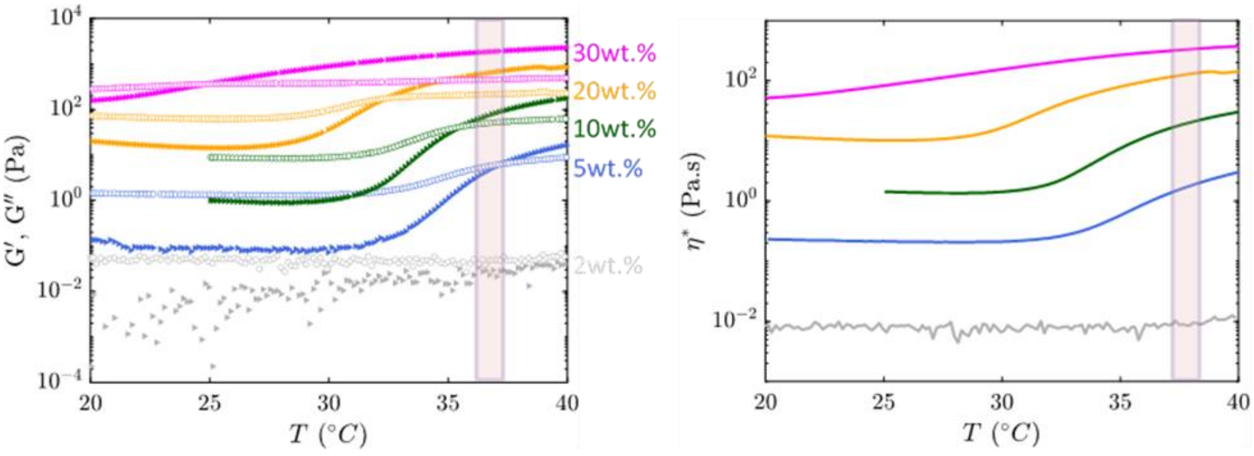

Fig. S3.

Measured modulus and complex viscosity during temperature sweep of LBL at different concentration. $n_{bb} = 553, n_L = 686, \phi_L = 0.2$. Transparent pink box represents 37°C. For the molecular structure and synthesis route, the reader is referred to Fig.2, fig. S5 and method section.

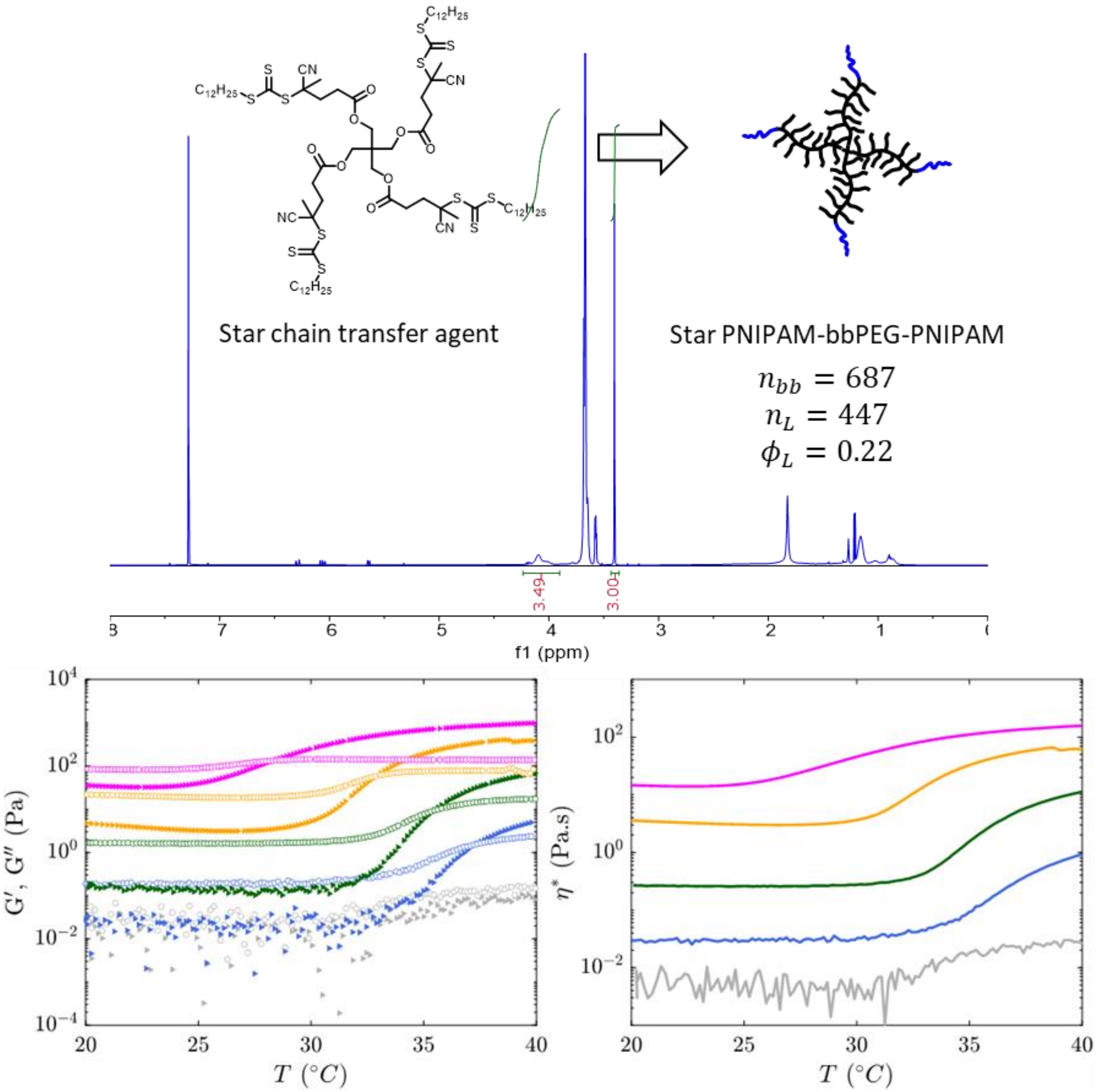

Fig. S4.

NMR spectra and measured modulus and complex viscosity during temperature sweep of star-LBL sample for different concentrations. $n_{bb} = 687, n_L = 447, n_{sc} = 9,\ \phi_L = 0.22$. Definition of lines is given in fig. S3. For synthesis route, the reader is referred to method section.

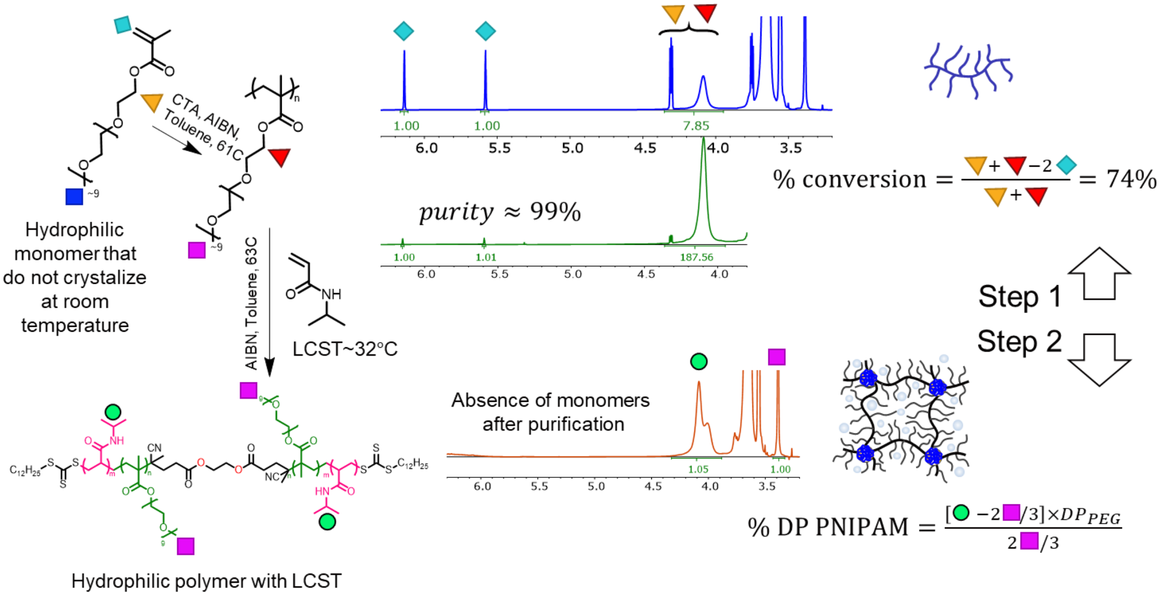

Fig. S5.

NMR of representative LBL, system 1 sample. Degree of polymerization for backbone and linear blocks was determined via 600MHz NMR Bruker at UNC core facility (LBL - system 1).

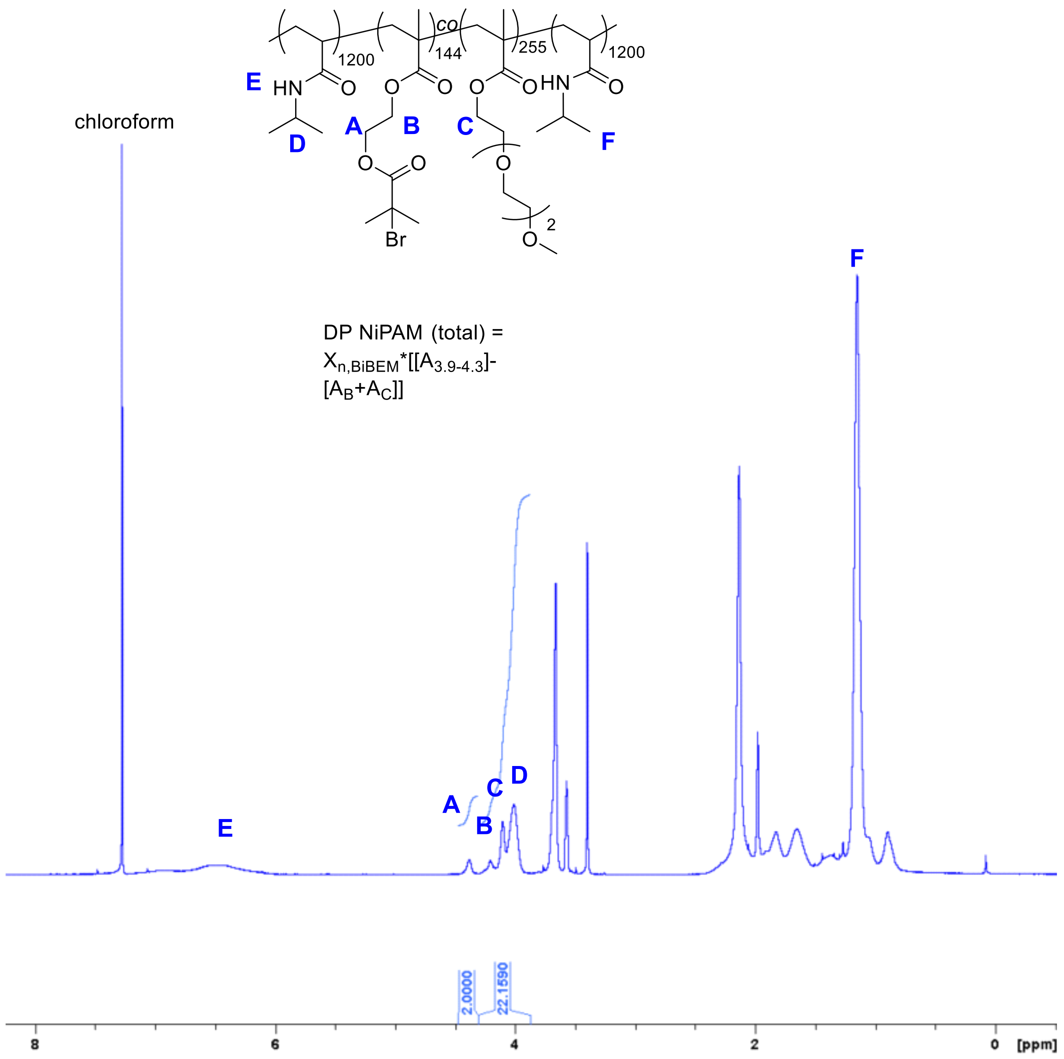

Fig. S6.

NMR of representative LBoBL, system 2 sample. ^{1}H NMR of $PNiPAM_{1200}$-b-P($BiBEM_{144}$-co-OEO_3MA_{255})-b-$PNiPAM_{1200}$. Spectra taken using a 500 MHz Bruker NMR in $CDCl_3$ (LBoBL - system 2).

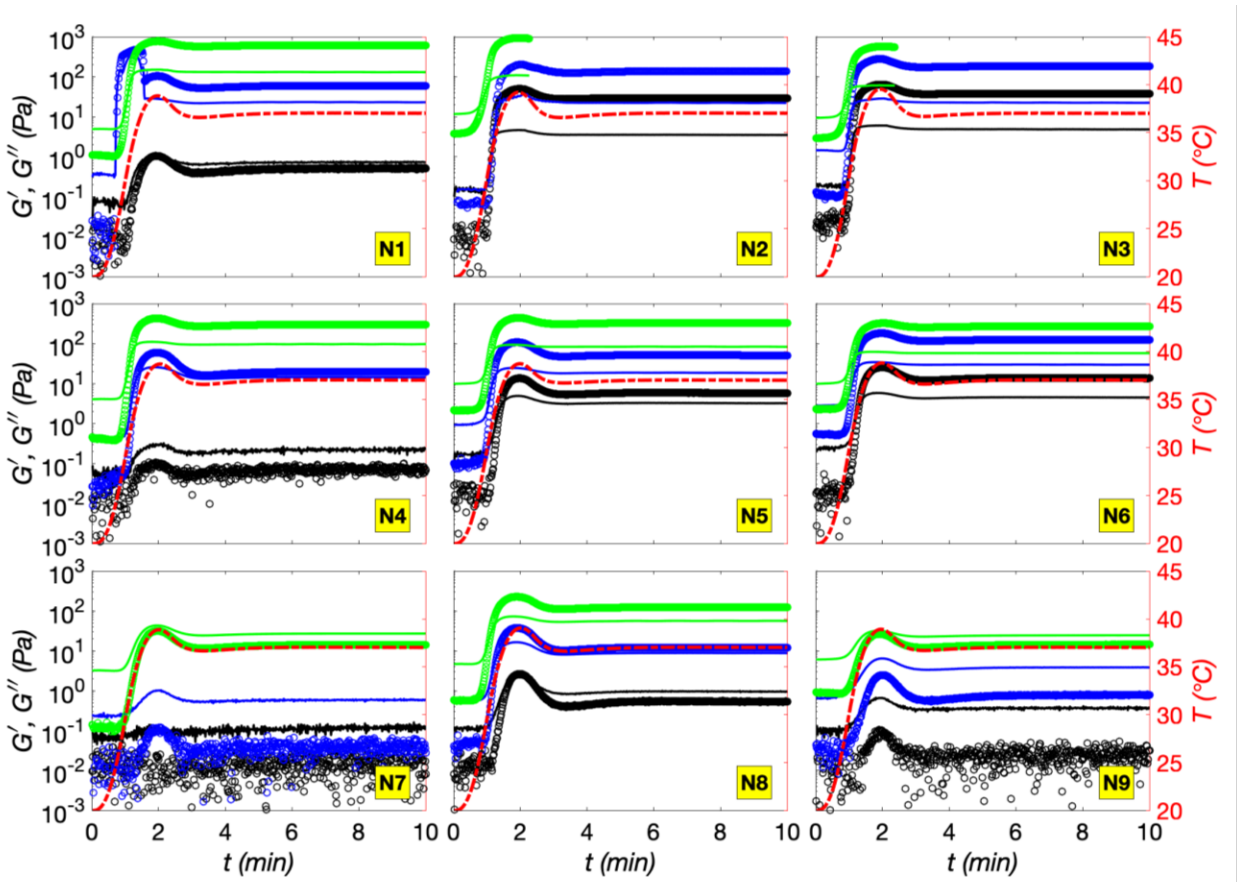

Fig. S7.

Prompt network formation (LBL). Samples (N1 – N9, follow the same order as fig. S6) at different water concentrations (5 (black), 10 (blue), 20 (green) wt.%). Hydrogels initially at rest were subjected to swift temperature ramp from 20 to 37°C and kept at 37°C for a period of 600 seconds. Hollow circles and solid lines indicate storage (G') and loss (G'') moduli, respectively. Temperature ramp is shown in red dashed line on right y-axis. TA instrument overshoots to about 39°C. All samples swiftly and closely follow changes made in the local temperature even during the overshoot period. Some of the samples (e. g., N7 did not gelate at the target temperature).

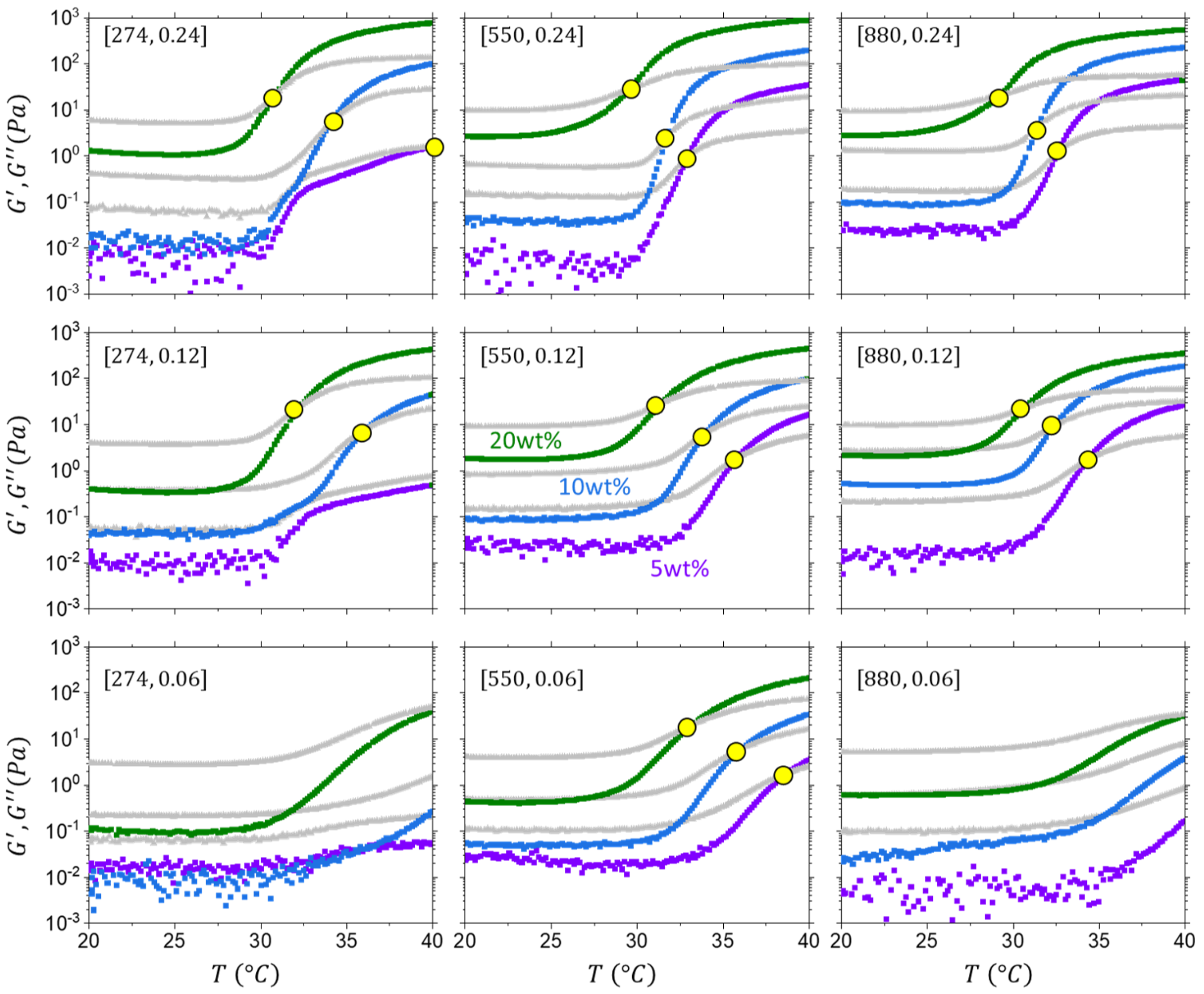

Fig. S8.

Gelation during heating cycle (LBL). Temperature sweep of samples with various degree of polymerization of backbone volume fraction of linear block. X-axis shows temperature varied from 20 to 40 °C at the rate of 1 (°C/min) and y-axis represents the storage ($G' - filled\ symbols$) and loss ($G'' - gray\ symbols$) modulus in Pa. The transition point from liquid to gel state occurs where storage moduli take over the loss moduli and is highlighted with yellow filled circles. The experiment is done at three different concentrations of 5 (purple), 10 (blue) and 20 (green) wt.%. Samples are represented with $[DP_{n_{bb}}, \phi_L]$ where some cases (e. g., [274,0.06]) do not show gelation behavior within the measurement window.

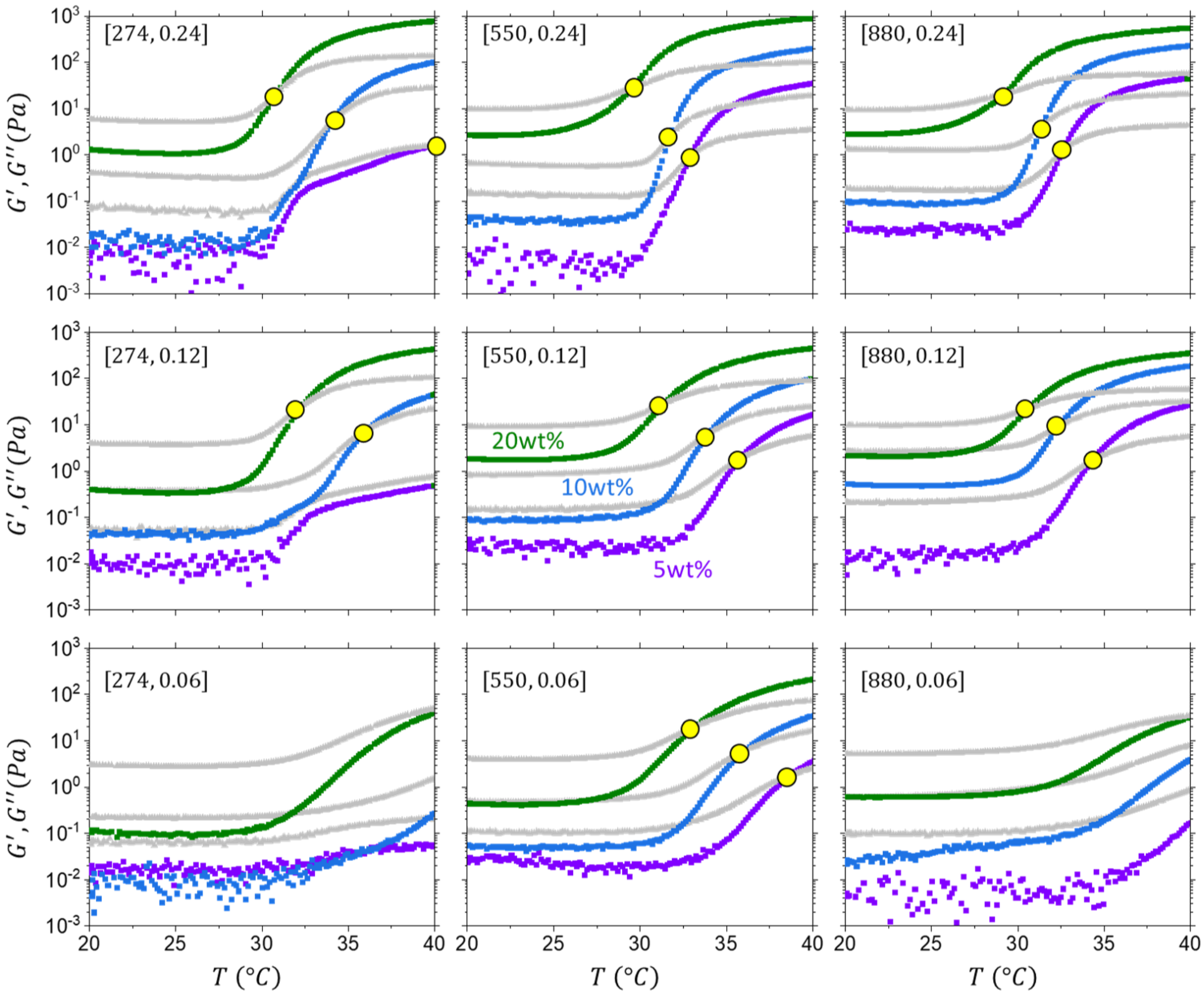

Fig. S9.

Cooling cycle and gel reversibility (LBL). Temperature sweep of samples with various degree of polymerization of back bone and linear block under cooling cycle. X-axis shows temperature varied from 40 to 20 $°C$ at the rate of 1 ($°C$ /min) and y-axis represents the storage ($G' - filled\ symbols$) and loss ($G'' - gray\ symbols$) modulus in Pa. The transition point from gel to liquid state is highlighted with yellow filled circles. The experiment is done at three different concentrations of 5 (purple), 10 (blue) and 20 (green) wt.%. Samples are represented with $[DP_{n_{bb}}, \phi_L]$.

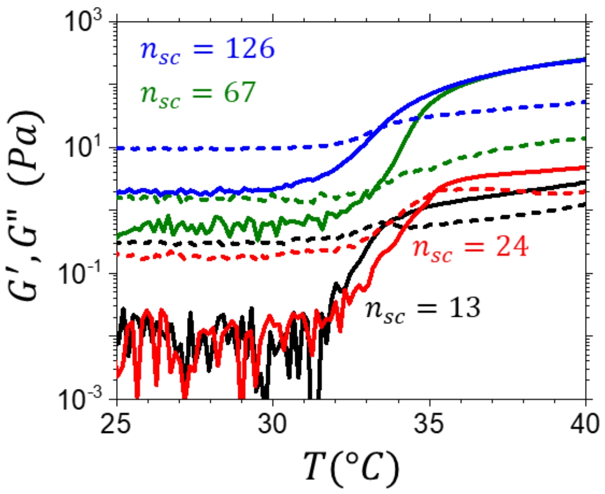

Fig. S10.

Temperature sweep of LBoBL (system 2) samples. Temperature sweep of samples with different n_{sc} at 10 wt.% concentration (Table 1), synthesized with three step combination of RAFT and ATRP (LBoBL - system 2) – X-axis shows temperature varied from 25 to 40 $°C$ at the rate of 1 ($°C$/min) and y-axis represents the storage ($G' - hollow\ symbols$) and loss ($G'' - lines$) modulus in Pa.

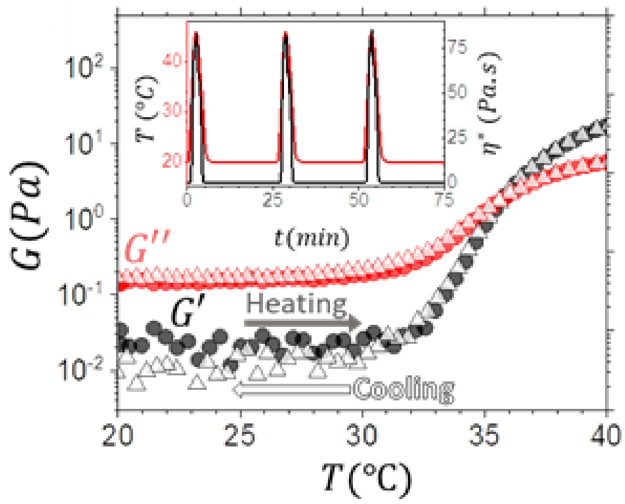

Fig. S11.

The gelation process is fully reversible upon heating-cooling cycles (LBL). Sample: 5 wt% solution of n_{bb} =550, ϕ_L =0.125 triblock. Temperature from 20 $°C$ to 40 $°C$ at 1 Hz. Inset: complex viscosity at 1 Hz over 75 minutes upon three consecutive heating-cooling cycles. Sample is heated rapidly to 45 $°C$ (closely following the instrument temperature overshoot to about 48 $°C$) followed by prompt cooling.

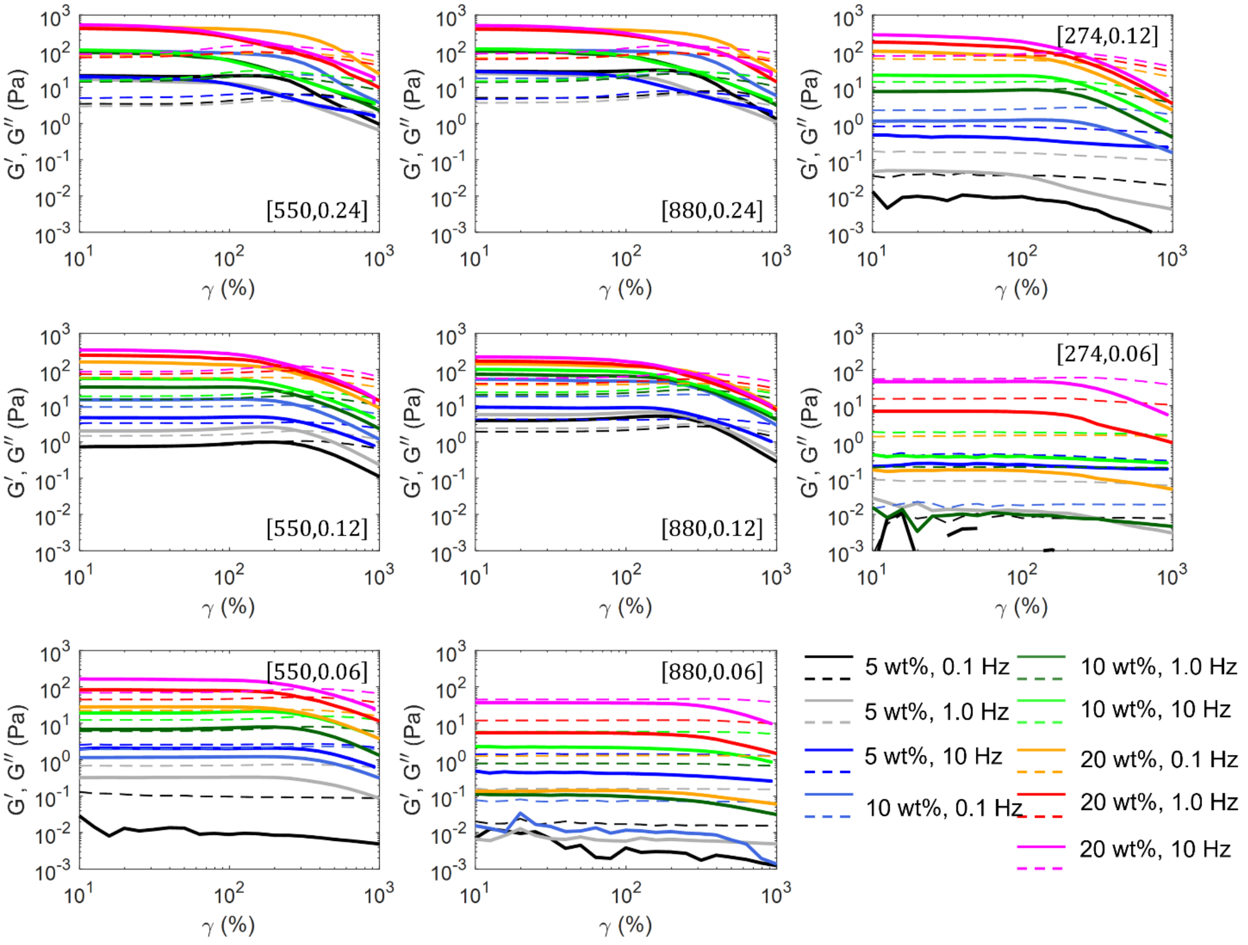

Fig. S12.

Strain endurance (forward cycle, LBL system 1). Strain sweeps of samples with different composition and concentration at physiological temperature of 37 (°C). Polymer is initially at gel state then shows liquid-like behavior under higher strains. Measurements were done at three frequencies of 0.1, 1 and 10 Hz. Strain applied from 10^{-2} to 10^{4} % (x-axis is limited to $[1, 1000]$%) and y-axis represents the storage ($G' - solid\ lines$) and loss ($G'' - dashed\ lines$) modulus in Pa. The transition point from gel to flow state occurs where loss moduli take over the storage moduli.

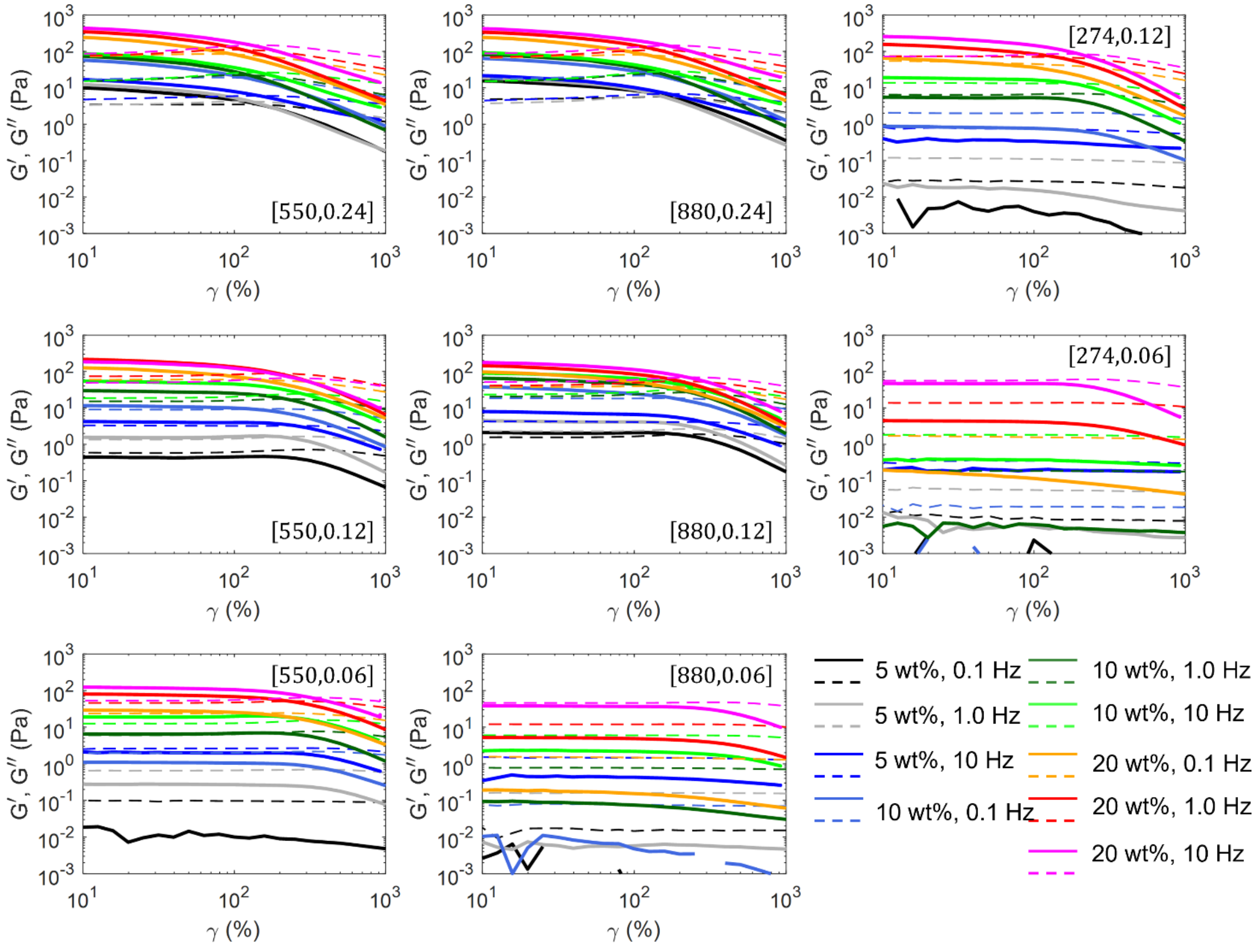

Fig. S13.

Strain endurance (reverse cycle, LBL system 1). Strain sweeps of samples with different composition and concentration at physiological temperature of 37 (°C). Polymer is initially at gel state then shows liquid-like behavior under higher strains. Measurements were done at three frequencies of 0.1, 1 and 10 Hz. Strain applied from 10^4 to 10^{-2} % (x-axis is limited to $[1, 1000]$%) and y-axis represents the storage ($G' - solid\ lines$) and loss ($G'' - dashed\ lines$) modulus in Pa. The transition point from gel to flow state occurs where loss moduli take over the storage moduli.

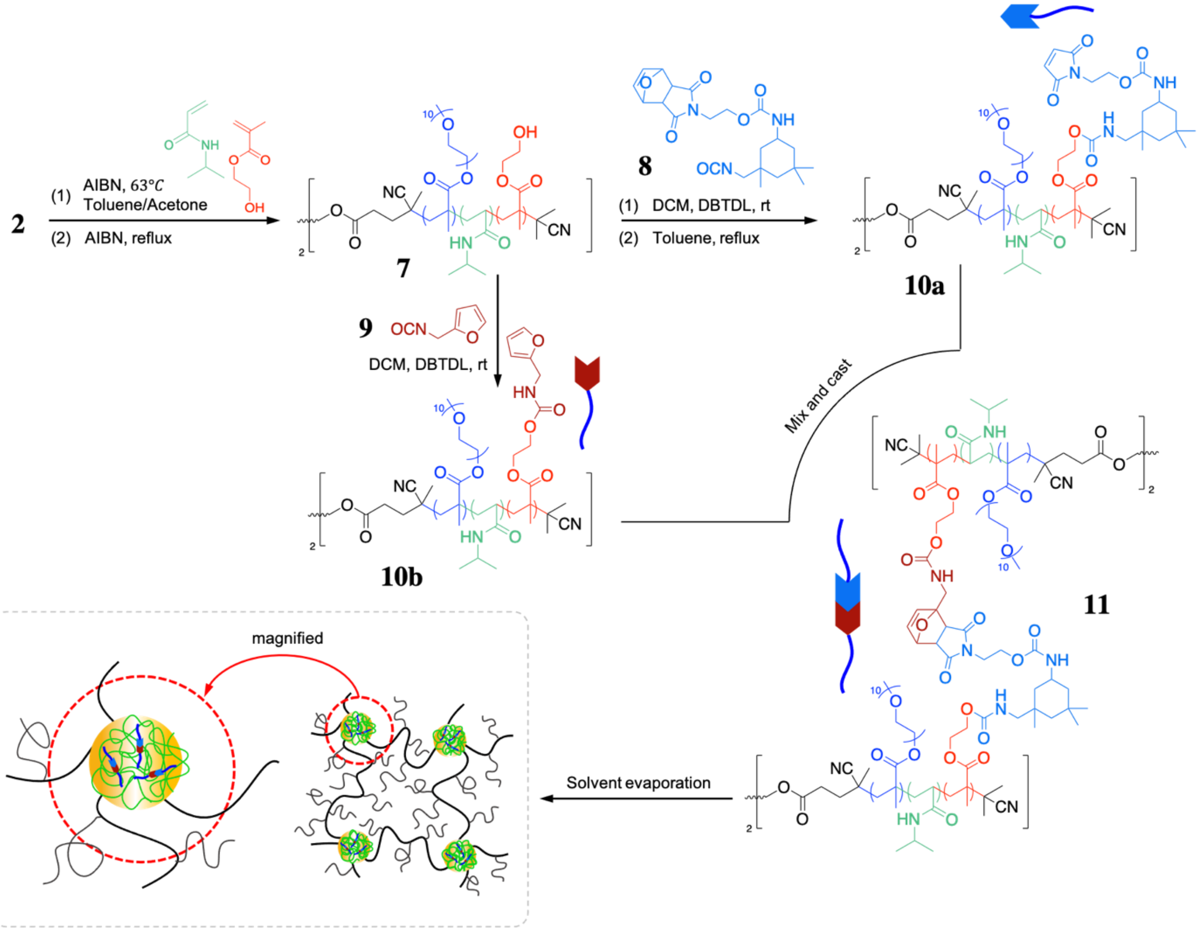

Fig. S14.

Dynamic crosslinking of tissue-mimetic elastomers (LBL system 1). Compound **2** is the PEG-brush bbCTA (Scheme 1 – system 1). The triblock is functionalized with copolymerization of HEMA (compound **7**, <5 mol%) during the second step of RAFT and is modified via post-polymerization. The LBL was under reflux for 8 hours with excess AIBN to remove CTA end groups and cap both chain ends. Compound **7** is treated with maleimide-isocyanate (**8**) and furfuryl isocyanate (**9**) Diels-Alder adducts in two separate reactions to yield **10a** and **10b**, respectively. The two polymers with functionalized moieties are then mixed to afford linear domain crosslinked bottlebrush elastomer (**11**). The dashed box schematically shows the phase separation of L-domains (and magnification on a single domain) at dry state followed by DA crosslinking among linear chains. Black, green and blue strands show the brush backbone, PNIPAM linear chains and functional groups, respectively.

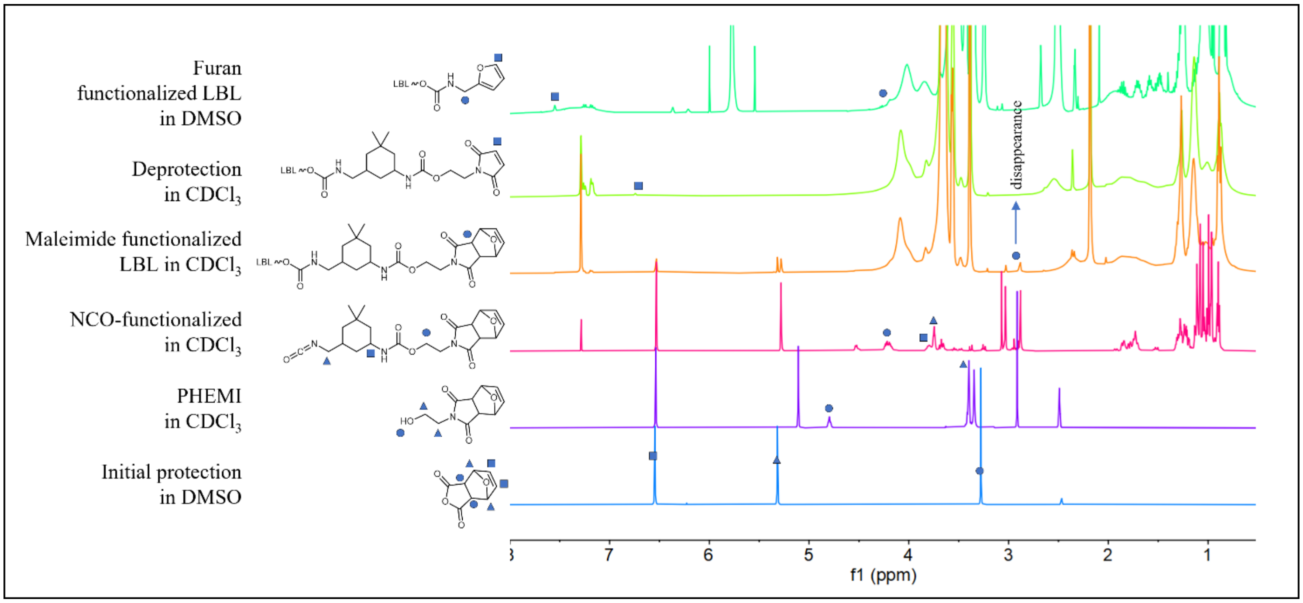

Fig. S15.

Preparation of DA moieties and attachment to the polymer chain. NMR spectroscopy of functionalization of linear domains of LBL (system 1) with maleimide and furan groups.

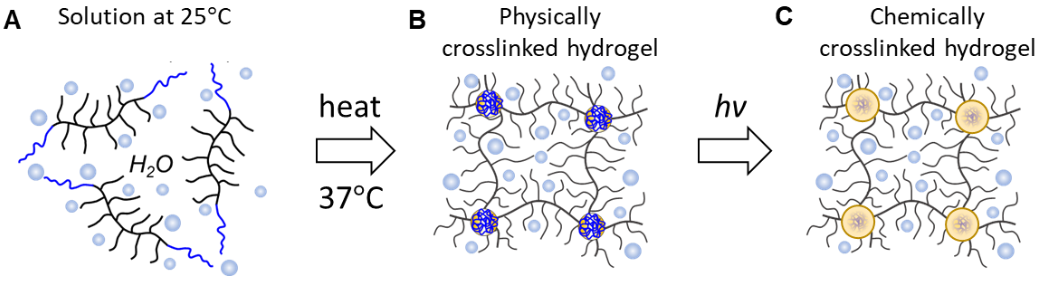

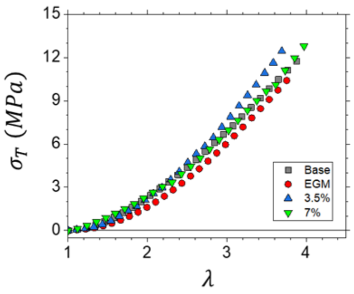

Sample	% crosslink	β	G (kPa)	E_0 (kPa)	σ_{max} (MPa)	λ_{fit}
Base	0	0.53	81.5	817.1	12.2	1.58
EGM	0	0.58	34.5	419.6	10.8	1.55
UV-1	3.5	0.61	39.1	553.5	12.6	1.52
UV-2	7.0	0.12	485.5	1750	13.3	2.21

Fig. S16.
UV crosslinking of LBL, system 1, hydrogels. UV cured hydrogels upon phase separation (gelation) above LCST. Base, EGM, UV-1 and UV-2 represent for different uniaxial stress-strain tests on PNIPAM-bbPEG-PNIPAM, RAFT end group removed PNIPAM-bbPEG-PNIPAM, 3.5% and 7% crosslinking, respectively.

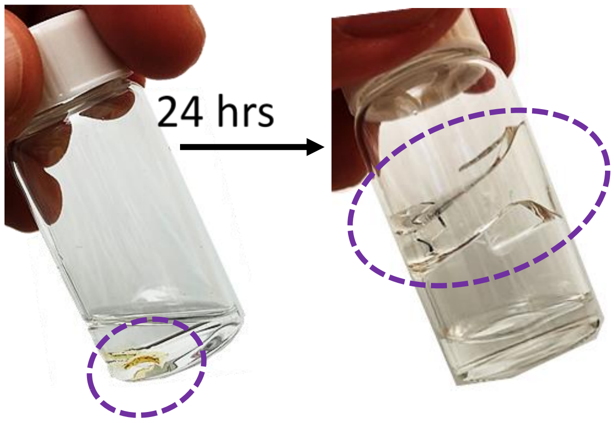

Fig. S17.
Swelling of DA-crosslinked LBL (system 1) elastomers. The residue of the stress-strain curve measurement, (Left) is place in scintillation vial in good solvent (acetone). After 24 hours the sample is fully swollen (equilibrium swelling ratio $Q = \frac{Weight_{gel}}{Weight_{dry}} = 13.8$).

To investigate the crosslinking effect on network structure, we conduct comparative tensile tests and SAXS measurements of physically and chemically crosslinked elastomers with the same LBL composition. The obtained stress-strain curves closely match at moderate elongation $\lambda < 2$, suggesting that the chemical crosslinking inside the L-domains do not perturb the overall network topology (fig. S18A). The small increase in stiffness is attributed to the decrease in the L-domain size due to introduction of the F and M groups to linear blocks (fig. S18B), resulting in a higher number of crosslink junctions at the same LBL composition.

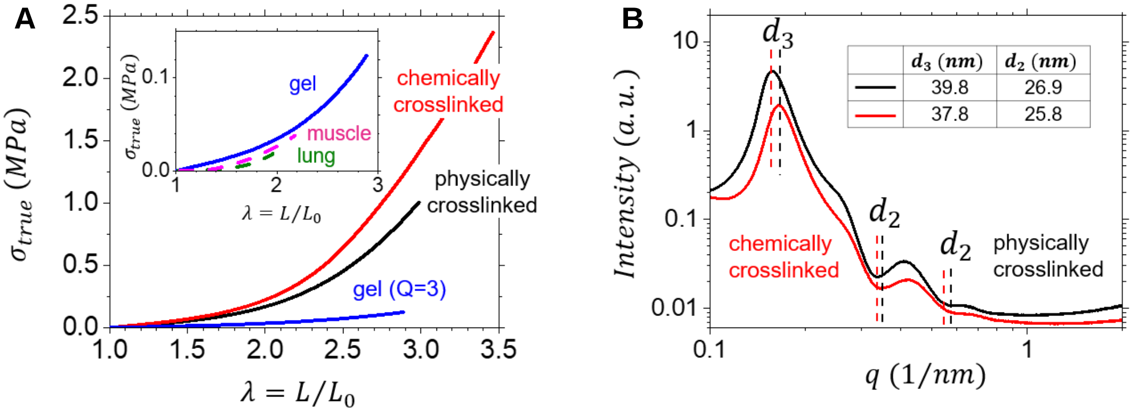

Fig. S18.

Permanent crosslinking mechanical properties (LBL, system 1). (A) Mechanical properties of physically crosslinked elastomer (black solid line), chemically crosslinked sample with identical chemical composition (red solid line) and swollen crosslinked sample with a swelling ratio of $Q = [m_p + m_w]/m_p = 3$. The LBL composition is: $n_{bb} = 607, n_L = 327, \phi_L = 0.19$. (inset) The crosslinked PNIPAM-bbPEG-PNIPAM hydrogel with $Q = 3$ (solid line) closely replicates the stress-elongation response of muscle and lung tissues (dashed lines). **(B)** 1D SAXS curves of physically and chemically crosslinked samples. Dashed lines mark the minima of the PNIPAM spheres form-factor (d_2) related to the diameter of the PNIPAM sphere (D) and the intersphere distance d_3.

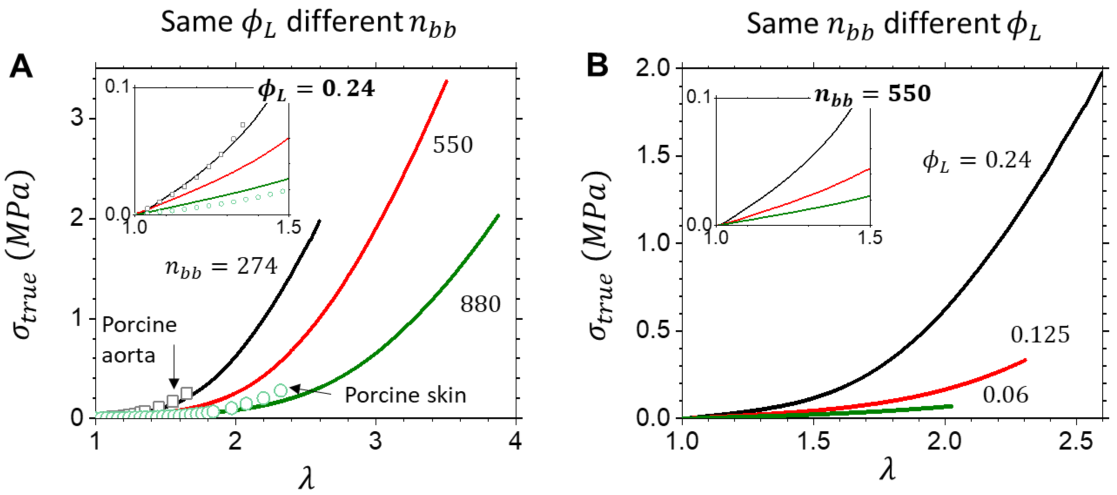

Fig. S19.

Mechanics of dry elastomers (LBL). (A) The true stress-elongation response of PNIPAM-bbPEG-PNIPAM LBL elastomers strongly depends on the B-block degree of polymerization ($\boldsymbol{n_{bb}}$) at same L-block volume fraction $\boldsymbol{\phi_L \cong 0.2}$. The LBL elastomers with $\boldsymbol{n_{bb}}$=274 and 880 closely match the deformation response of porcine aorta and skin tissues, respectively (hollow symbols). **(B)** Effect of the L-block volume fraction $\boldsymbol{\phi_L}$ on the stress-elongation response at constant backbone length with $\boldsymbol{n_{bb} = 550}$. The increase in $\boldsymbol{\phi_L}$ enhances both the strain-stiffening behavior and strength ($\boldsymbol{\sigma_{max}}$).

Theoretical analysis of gelation temperature T_{gel}

The effect of the PNIPAM block DP n_L on the gelation temperature T_{gel} could be rationalized on the basis of scaling arguments. At low concentrations of triblock copolymer in solution, gelation/network formation might be associated with micellization of PNIPAM-bbPEG-PNIPAM, triggered by temperature-induced condensation of linear (PNIPAM) blocks. An increase in the solution temperature T leads to aggregation of PNIPAM blocks in the cores of spherical micelles, interconnected by bridging bbPEG blocks. The number of PNIPAM blocks in the core of micelle (the aggregation number Q) can be estimated using the scaling theory of polymer micelles.

Due to low content of PNIPAM in the copolymer micelles are starlike, with thickness of PEG corona larger than the core size. In the vicinity of $T_{LCST} \approx 32°C$ for pure PNIPAM solution, the PEG monomers have positive but small second virial coefficient, which indicates that central PEG block is close to theta-solvent conditions. In this case, the aggregation number Q in starlike micelle is regulated by the length n_L of the condensed PNIPAM block and also depends on the solution concentration c.

The scaling model of spherical micelles predicts three different regimes of micelles formed by AB diblock copolymer with linear blocks. We assimilate n_L of PNIPAM to N_B of core-forming block B, and implement theta-solvent conditions for bbPEG (soluble block A). Because side chains length in the bbPEG block is small ($n_{sc} = 9$), in scaling terms it can be treated as linear, and $n_{bb}/2$ of bbPEG block is related to N_A of block A as $n_{bb} \sim N_A\, n_{sc}^{-1} \sim N_A$. In a dilute solution of micelles (below $c^* \sim n_L^{1/5} n_{bb}^{-1/2}$), the aggregation number Q does not depend on c, and depends on the parameters of the corona (bb) block only logarithmically. With omitted logarithmic prefactor, the power law dependence for micelle aggregation number is given by

$$Q \sim \gamma^{\frac{6}{5}} \left(\frac{n_L}{\phi}\right)^{\frac{4}{5}} \qquad (1)$$

where γ is surface tension (surface free energy of unit area at the core/corona interface, expressed in units of $k_B T$), and ϕ is volume fraction of PNIPAM monomers in the core of micelle.

At concentrations $c^{**} > c > c^*$ with $c^{**} \sim n_L^{-1/5}$ the omitted in eq 1 prefactor incorporates ratio (c/c^*) indicating logarithmically weak increase in Q, while at $c > c^{**}$ the scaling dependence for Q changes to

$$Q \sim n_L \phi c \qquad (2)$$

and micelles start to grow with increasing solution concentration, c, reaching its maximal size in melts with $\phi \sim c \sim 1$.

Near T_{LCST}, the volume fraction ϕ of PNIPAM monomers in the core is relatively low. Like in the case of infinitely large globule with positive third virial coefficient of monomer-monomer interactions, ϕ be specified via the second virial coefficient of binary monomer-monomer contacts,

$v(T) \sim -(T - T_{LCST})/T$, as $\phi \sim -v$. Notably, $v(T)$ accounts for the temperature-dependent hydration of PNIPAM monomeric units. In water solution of PNIPAM macromolecules with infinite DP, T_{LCST} is specified by $v(T_{LCST}) = 0$. However, the presence of PEG in PNIPAM-bbPEG-PNIPAM triblock copolymer can shift sign reversal of $v(T)$ to temperature $T = T_0 \neq T_{LSCT}$, to change $v(T)$ to $v'(T) \sim -(T - T_0)/T = -\tau$, and specify $\phi \sim \tau$. Here, superscript in v' signifies the second virial coefficient of PNIPAM monomers incorporated in the triblock copolymer. The surface tension γ at the surface of PNIPAM core is then linked to τ as $\gamma \sim \tau^2$.
By substituting $\gamma \sim \tau^2$ and $\phi \sim \tau$ in eqs 1 and 2, one finds

$$Q \sim \begin{cases} \tau^{8/5} n_L^{4/5} & c < c^{**} \sim \tau^{3/5} n_L^{-1/5} \\ \tau n_L\, c & c > c^{**} \end{cases} \qquad (3)$$

If the gelation threshold temperature, T_{gel}, is associated with onset of micellization in the solution, then formation of micelle with $Q \sim 1$ specifies $\tau_{gel} = \left(T_{gel} - T_0\right)/T_{gel}$ as

$$\tau_{gel} \sim \begin{cases} n_L^{-1/2} & c < c^{**} \sim n_L^{-1/2} \\ n_L^{-1} c^{-1} & c > c^{**} \end{cases} \qquad (4)$$

To quantitatively compare the experimental $T_{gel}(n_L)$ to eq 4, one should estimate T_0 corresponding to $v'(T_0) = 0$. In the mean-field framework, $v'(T)$ can be approximated as

$$v'(T) = v(T) + \Delta v(T) \qquad (5)$$

Here, $v(T)$ is the second virial coefficient of monomer-monomer interactions in the solution of infinitely long PNIPAM macromolecules at temperature T (in the absence of PEG), while $\Delta v(T)$ is the contribution due to plausible interactions between PNIPAM and PEG monomer units. By expanding $v(T)$ in Taylor series with retention of only linear in $(T_0 - T_{LCST})$ term one finds

$$v'(T_0) \approx v(T_{LCST}) + \left(\frac{dv}{dT}\right)_{T_{LCST}} (T_0 - T_{LCST}) + \Delta v(T_0) \qquad (6)$$

Notably, $v(T_{LCST}) = 0$, while the derivative $\left(\frac{dv}{dT}\right)_{T_{LCST}}$ is negative.
By using the condition $v'(T_0) = 0$, one finds

$$T_0 - T_{LCST} = -\frac{\Delta v(T_0)}{\left(\frac{dv}{dT}\right)_{T_{LCST}}} \sim \Delta v(T_0) \qquad (7)$$

If $\Delta v(T_0)$ is positive, then $T_0 > T_{LSCT}$, while if it is negative, then $T_0 < T_{LSCT}$. A negative value of $\Delta v(T_0)$ corresponds to an affective attraction/complexation between PNIPAM and PEG below T_{LCST}. Because hydrogen bonding is energetically favorable at low temperatures, attraction could (hypothetically) arise if PNIPAM and PEG form hydrogen bonds with the same water molecule or between PEG backbone and pendant groups of PNIPAM. As it follows from Fig. 4C, gelation temperature T_{gel} for several experimentally investigated samples is found below T_{LCST} of PNIPAM ($32°C$), indicating a possible shift in $(T_0 - T_{LCST}) < 0$. To the best of our knowledge there is no theoretical model relating T_0 to composition of PNIPAM-bbPEG-PNIPAM block copolymer or its concentration in the solution, and we implement T_0 as an adjustable parameter. In fig. S20, we present $\frac{T_{gel}-T_0}{T_{gel}}$ as a function of n_L for three solution concentrations (5, 10, and 20 wt.%) in $log-log$ coordinates. The data for all three solution concentrations collapsed on a master curve if values of T_0 were adjusted as 31.5 $°C$ (5 wt.%), 29 $°C$ (10 wt.%), and 27 $°C$ (20 wt.%). The slope of master curve $-1/2$ was in good agreement with the theoretical predictions in eq. 4 for the interval of solution concentrations $c < c^{**}$. Therefore, gelation and micellization of PNIPAM-bbPEG-PNIPAM block copolymer could be linked due to plausible association of PNIPAM and PEG monomer units below T_{LCST}.

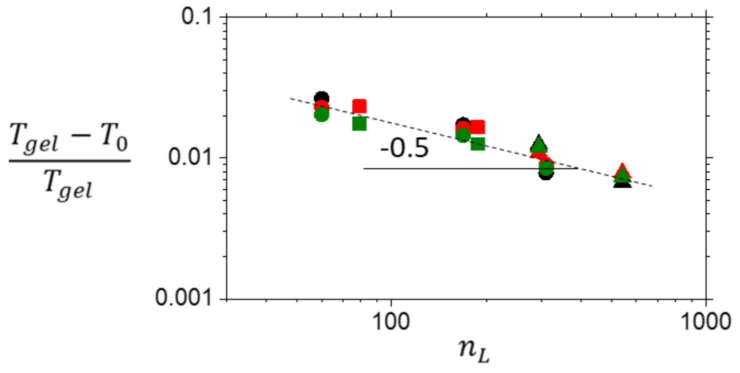

Fig. S20.

Theoretical prediction of gel temperature with change in n_L. Ratio $\frac{T_{gel}-T_0}{T_{gel}}$ as a function of DP (n_L) of linear PNIPAM block in PNIPAM-bbPEG-PNIPAM block copolymer. Symbols follow legends in Fig. 4D. Values of T_0 were adjusted as $30.5°C$ (5 wt.%, black symbols), $29°C$ (10 wt.%, red symbols), $27°C$ (20 wt.%, green symbols). Straight line has slope -1/2.

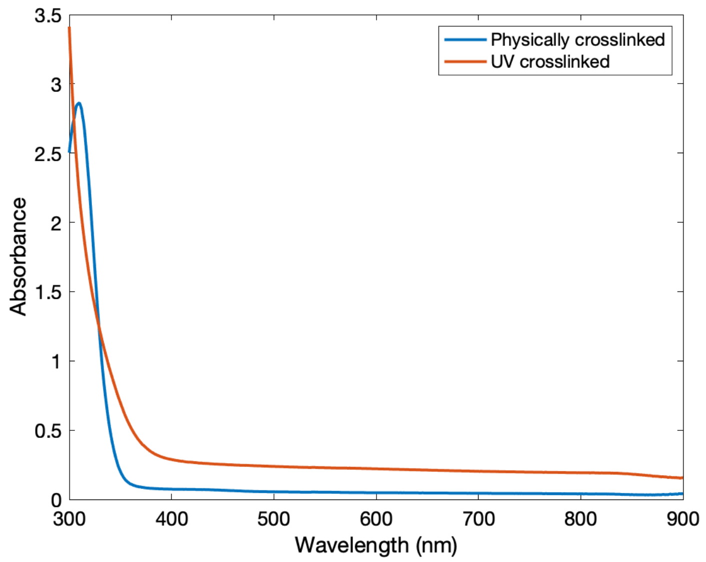

Fig. S21.

Ultraviolet–visible (UV–Vis) spectroscopy. UV-Vis of PNIPAM-bbPEG-PNIPAM samples in physically and chemically crosslinked states. Both samples show similar spectra between the range of 350 to 900 nm, indicative of optical transparency before and after UV-crosslinking.

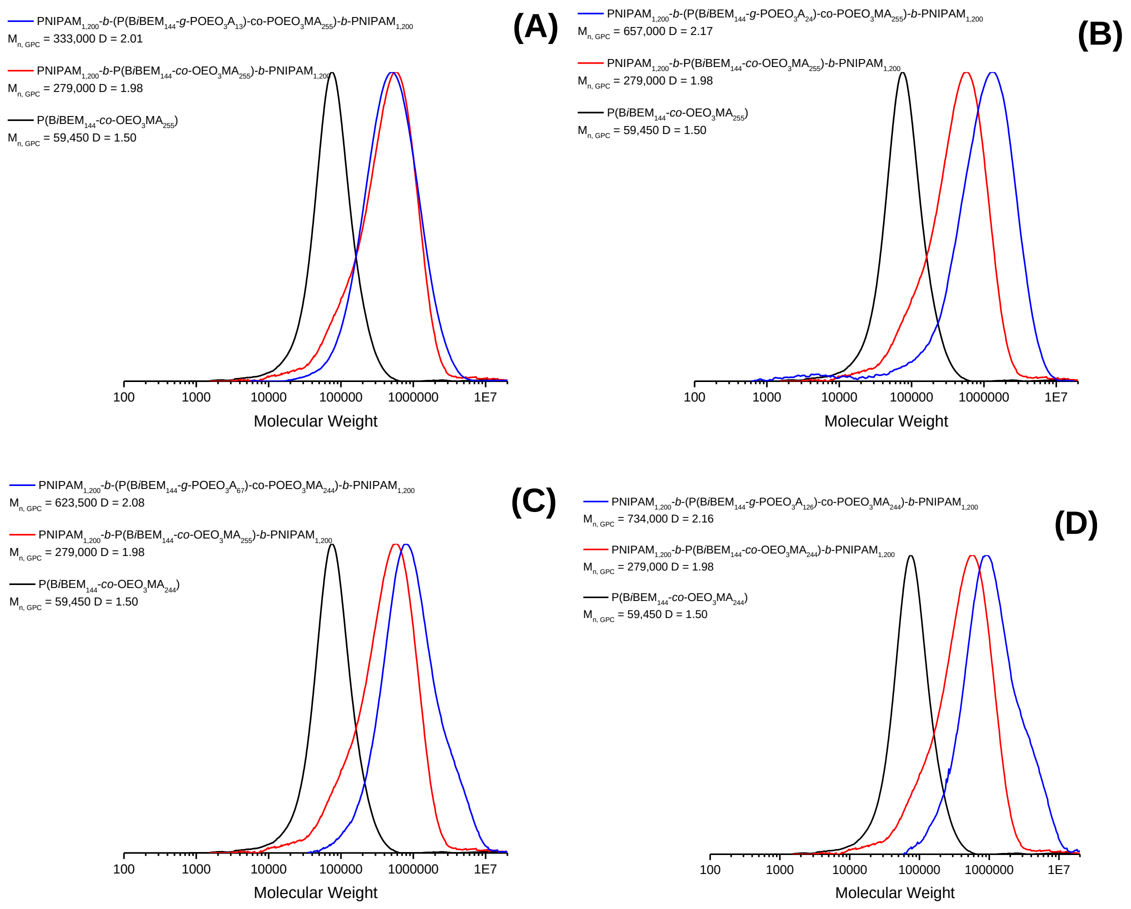

Fig. S22.

GPC spectra of LBoBL samples. n_{sc} = (A) 13; (B) 24; (C) 67; and (D) 126. Molecular weight is given relative to linear PMMA standards.

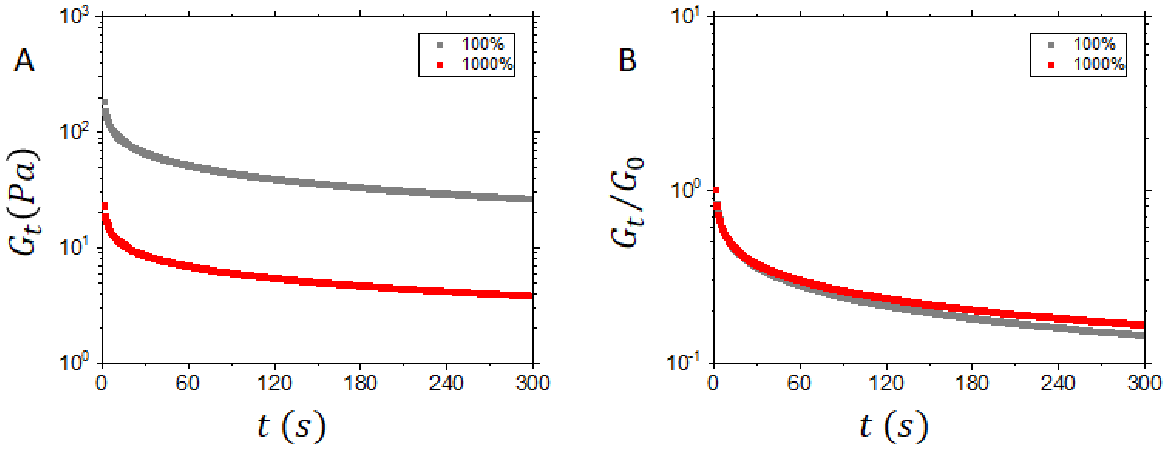

Fig. S23.

Stress-relaxation of LBL sample. A) Modulus and B) normalized modulus relaxation response of PNIPAM-bbPEG-PNIPAM ($n_{bb} = 600, n_L = 200, \phi_L = 0.13, T_{gel} = 33.4°C$) was measured by applying 100% strain<γ_{crt} (gray) and 1000% strain>γ_{crt} (red) and monitoring its relaxation over time (300s) at 37°C. Upon stress network dissociates and reassembles over time. Under both strains, sample show similar timescale recovery.

Table S1.
Rheological characteristics of hydrogels at different LBL compositions and concentrations.

n_{bb} [1)]	n_L [2)]	ϕ_L [3)]	$wt.\%$ [4)]	$T_{gel}(°C) - Heat$	$G_{gel}(Pa) - Heat$	$T_{gel}(°C) - Cool$	$G_{gel}(Pa) - Cool$	$G_{37}(Pa)$	$Hysteresis\ (°C)$	$\eta^*(20.5\ °C)$ Pa.s	$\eta^*(37\ °C)$ Pa.s
274	34	0.05	5	NaN	NaN	NaN	NaN	NaN	NaN	NaN	NaN
			10	NaN	NaN	NaN	NaN	NaN	NaN	NaN	NaN
			20	NaN	NaN	NaN	NaN	NaN	NaN	NaN	NaN
	79	0.12	5	NaN	NaN	NaN	NaN	NaN	NaN	NaN	NaN
			10	36.2	8.3	36.2	8.1	15.2	0.03	0.06	3.08
			20	32.4	26.7	32.0	24.0	283.9	0.33	0.62	47.41
	188	0.24	5	39.9	1.6	39.8	1.5	0.8	0.04	0.01	0.22
			10	34.1	5.5	33.8	4.7	51.2	0.27	0.06	8.87
			20	30.8	21.5	30.0	15.5	583.1	0.81	0.93	94.96
550	60	0.05	5	38.7	1.8	38.7	1.8	0.6	0.03	0.02	0.18
			10	36.1	6.1	36.1	6.1	12.0	0.00	0.08	2.38
			20	33.2	20.6	33.0	19.8	119.1	0.26	0.64	20.92
	169	0.12	5	35.8	1.9	35.8	1.9	5.2	0.05	0.02	0.96
			10	34.0	6.5	34.0	6.4	47.9	0.03	0.13	8.15
			20	31.4	29.5	31.0	27.4	316.6	0.40	1.47	51.96
	310	0.2	5	32.9	0.9	32.6	0.8	21.3	0.31	0.02	3.42
			10	31.7	2.6	31.1	2.2	132.3	0.63	0.10	21.18
			20	29.6	27.9	27.1	20.7	713.1	2.49	1.59	114.37
880	113	0.06	5	NaN	NaN	NaN	NaN	NaN	NaN	NaN	NaN
			10	NaN	NaN	NaN	NaN	NaN	NaN	NaN	NaN
			20	NaN	NaN	NaN	NaN	NaN	NaN	NaN	NaN
	294	0.13	5	34.3	1.7	34.1	1.6	12.1	0.20	0.03	2.03
			10	32.4	9.2	32.1	8.6	119.8	0.24	0.43	19.57
			20	30.7	23.1	30.3	22.4	267.3	0.35	1.57	43.42
	542	0.22	5	32.6	1.2	32.2	1.0	30.5	0.33	0.03	4.89
			10	31.4	4.2	31.0	4.2	169.8	0.40	0.21	27.19
			20	29.2	20.5	28.4	19.6	454.1	0.80	1.53	72.76

[1)] Degree of polymerization (DP) of PEG bottlebrush backbone, [2)] DP of PNIPAM linear block. [3)] Volume fraction of linear domains $\frac{V_{Lin}}{V_{Lin}+V_{brush}}$, [4)] Solution concentration $\frac{m_{polymer}}{m_{polymer}+m_{water}}$, $T_{gel} - Heating$ and $T_{gel} - Cooling$: transition temperatures measured upon heating (fig. S8) and cooling (fig. S9) cycles, respectively. $G_{gel} - Heating$ and $G_{gel} - Cooling$ are the corresponding storage moduli at $T = T_{gel}$. G_{37} is storage modulus at $T = 37$℃ (heating cycles, fig. S8). $\eta^*_{20.5}$ and η^*_{37} are complex viscosity at 20 $°C$ and 37 $°C$, respectively.

Table S2.

Mechanical properties of elastomers.

n_{bb}	n_L	ϕ_L	β	$G\ (kPa)$	λ_{fit}	E_0, fit	E_0, exp	$\sigma_{max}\ (MPa)$
**System 1**								
274	34	0.05	0.24	9.4	1.70	42.1	40.8	0.06
	79	0.12	0.35	12.8	1.77	72.2	70.8	0.33
	188	0.24	0.51	15.8	1.64	148.3	142.2	1.25
550	60	0.05	0.19	8.2	2.08	32.9	34.6	0.56
	169	0.12	0.21	8.7	1.93	36.5	38.3	1.08
	310	0.2	0.36	15.8	1.98	93.6	96.8	3.21
880	113	0.05	0.11	5.4	2.04	18.9	20.9	0.10
	294	0.13	0.17	7.8	1.65	30.9	32.5	1.01
	542	0.22	0.26	9.9	2.17	45.4	49.0	1.97
**System 2**								
n_{bb}	n_L	n_{sc}[i)]	ϕ_L	β	$G\ (kPa)$	λ_{fit}	$E_0\ (kPa)$	$\sigma_{max}\ (MPa)$
399	1200	13	0.38	0.68	NA	1.4	203.2	0.1
		24	0.28	0.6	NA	1.5	79.6	1.1
		67	0.13	0.85	NA	1.15	127.6	0.38

n_{bb} : degree of polymerization of backbone brush. n_L : degree of polymerization of linear domains (PNIPAM). ϕ_L : volume fraction of linear chains. β: strain-stiffening (or firmness) parameter acquired from fitting the true stress verses strain curves. $E = 3G$: structural modulus acquired from the fitting equation. λ_{fit}: fitting intervals used to fit the stress-strain curves. E_0, fit and E_0, exp are Young's modulus from fitting stress-strain curves with the equation of state (eq S8) and experimental measurements as a slope at zero strain, respectively. σ_{max} : true stress at break.

Table S3.

Small angle X-ray analysis of PNIPAM-bbPEG-PNIPAM elastomers

n_{bb} [1)]	n_L [1)]	ϕ_L [1)]	x-link [2)]	E_0 (kPa) [3)]	β [4)]	d_3 (nm) [5)]	D (nm)[6)]	RSD[7)]	Q [8)]
304	157	0.10	no	223	0.29	29.3	19.1	0.19	136
560	369	0.12	no	99.6	0.23	39.8	26.9	0.11	162
560	900	0.26	no	2300	0.14	57.3	35.2	0.17	149
560	369	0.12	yes	279	0.43	37.8	25.8	0.12	144

[1)]Architectural parameters of PNIPAM-bbPEG-PNIPAM triblock copolymers. [2)]One sample ($n_{bb} = 560, n_L = 369$) was chemically crosslinked with 1.5 mol% is Diels-Alder links within the PNIPAM domains. [3)]Young's modulus parameter. [4)]Firmness parameter characterizing the strain-stiffening response. [5)]Position of the main interference peak corresponding to the distance between the PNIPAM domains. [6)]Diameter of PNIPAM spherical domains is determined from the fit of the SAXS curves to the form-factor of spheres. [7)]Relative standard deviation of d_2. [8)]Aggregation number determined as $Q = \pi d_2^3/(6 n_L v_L)$, where $v_L = M_0/(\rho N_{Av}) = 0.171\ nm^3$ is volume of NIPAM monomer.

Table S4.

Critical strain at which storage moduli equates loss moduli extracted from figs. S12 and S13. Measurements were done at three frequencies of 0.1, 1, and 10 Hz for Forward (increase in strain from 10^{-2} to 10^{4}) and Reverse strain, γ (%) (decrease from 10^{4} to 10^{-2}). For the description of headers refer to table S1.

n_{bb}	n_L	ϕ_L	$wt.\%$	γ (%)					
				**0.1 Hz**		**1.0 Hz**		**10 Hz**	
				**Forward**	**Reverse**	**Forward**	**Reverse**	**Forward**	**Reverse**
550	310	0.2	5	386.3	150.0	312.6	128.2	189.7	147.8
			10	468.0	120.7	265.6	126.2	186.4	146.0
			20	682.5	98.9	299.6	115.0	217.9	131.2
880	542	0.22	5	473.5	173.6	359.6	152.9	240.3	169.4
			10	566.7	149.6	360.2	152.6	284.8	169.7
			20	629.9	124.0	498.5	146.1	282.9	166.1
274	79	0.12	5	NA	NA	NA	NA	NA	NA
			10	NA	NA	115.5	NA	214.3	180.0
			20	170.9	36.6	194.0	142.5	222.6	200.3
550	169	0.12	5	15.7	NA	293.4	204.0	307.8	254.9
			10	288.6	131.7	299.1	222.7	306.8	289.6
			20	370.7	147.5	297.2	195.7	271.0	248.7
880	294	0.13	5	384.5	201.8	380.8	277.1	348.6	289.8
			10	414.1	183.5	354.7	237.3	301.7	271.6
			20	525.0	158.0	313.8	201.4	263.9	228.7
274	34	0.05	5	16.1	NA	NA	NA	NA	NA
			10	NA	NA	NA	NA	NA	NA
			20	NA	NA	NA	NA	NA	NA
550	60	0.05	5	NA	NA	NA	NA	NA	NA
			10	NA	NA	234.0	172.1	347.0	327.6
			20	224.8	127.8	299.7	233.2	338.3	320.4
880	113	0.06	5	NA	NA	NA	NA	NA	NA
			10	NA	NA	NA	NA	NA	NA
			20	NA	NA	NA	NA	NA	NA

Table S5.

Critical (storage) modulus at corresponding critical strain extracted from figs. S12 and S13. Measurements were done at three frequencies of 0.1, 1, and 10 Hz for Forward (increase in strain from 10^{-2} to 10^{4}) and Reverse (strain decrease from 10^{4} to 10^{-2}). For the description of headers refer to table S1.

n_{bb}	n_L	ϕ_L	$wt.\%$	$G\ (Pa)$					
				**0.1 Hz**		**1.0 Hz**		**10 Hz**	
				**Forward**	**Reverse**	**Forward**	**Reverse**	**Forward**	**Reverse**
550	310	0.2	5	6.4	3.8	6.8	3.4	4.2	6.0
			10	20.5	19.2	28.5	18.7	22.2	26.5
			20	65.8	80.7	142.0	82.8	110.8	147.8
880	542	0.22	5	8.0	5.6	7.8	5.7	5.9	6.8
			10	23.0	19.8	28.1	21.3	24.4	28.2
			20	74.9	71.3	138.9	78.8	108.5	144.5
274	79	0.12	5	NA	NA	NA	NA	NA	NA
			10	NA	8.5	14.5	NA	NA	13.2
			20	52.8	73.6	88.7	50.7	69.2	84.6
550	169	0.12	5	0.7	2.0	3.5	NA	1.6	3.2
			10	10.3	18.3	25.1	9.3	17.5	24.1
			20	54.1	89.2	124.8	58.9	88.1	66.5
880	294	0.13	5	2.6	3.1	4.4	1.9	2.7	4.1
			10	18.9	27.0	36.3	18.4	26.6	33.4
			20	35.5	54.0	80.6	39.0	54.1	68.8
274	34	0.05	5	0.0	NA	NA	NA	NA	NA
			10	NA	NA	NA	NA	NA	NA
			20	NA	NA	NA	NA	NA	NA
550	60	0.05	5	NA	NA	NA	NA	NA	NA
			10	NA	7.7	15.7	NA	6.9	15.4
			20	23.8	52.2	88.4	24.2	51.2	64.5
880	113	0.06	5	NA	NA	NA	NA	NA	NA
			10	NA	NA	NA	NA	NA	NA
			20	NA	NA	NA	NA	NA	NA

Table S6.

Measure of gel reversibility under strain. Gel storage modulus at $\gamma = 10\%$ was extrapolated from strain sweep curves (figs. S12 and S13). G_F and G_R indicate the storage modulus extrapolated from forward and reverse cycles at $\gamma = 10\%$, respectively. For the description of headers refer to table S1.

n_{bb}	n_L	ϕ_L	$wt.\%$	$G_F - G_R$ at $\gamma = 10\%$					
				**0.1 Hz**		**1.0 Hz**		**10 Hz**	
				**Forward**	**Reverse**	**Forward**	**Reverse**	**Forward**	**Reverse**
550	310	0.2	5	20.9	16.5	19.6	9.6	11.8	16.2
			10	102.9	90.9	106.0	54.9	70.3	83.8
			20	482.8	415.5	526.6	231.7	332.8	419.9
880	542	0.22	5	27.7	24.0	27.0	15.1	18.2	21.1
			10	110.0	98.5	114.2	61.8	79.2	89.5
			20	438.3	401.6	506.2	233.7	326.7	413.1
274	79	0.12	5	0.0	0.1	0.5	0.0	0.0	0.3
			10	1.2	7.7	21.6	0.9	5.5	19.0
			20	98.9	175.6	280.5	64.0	151.6	253.0
550	169	0.12	5	0.7	2.0	4.7	0.5	1.6	4.2
			10	14.7	32.9	56.9	11.5	29.5	53.5
			20	162.6	246.9	347.2	123.4	212.1	182.2
880	294	0.13	5	3.9	5.7	9.1	2.1	4.4	7.9
			10	53.5	73.6	100.7	36.8	64.0	89.4
			20	143.5	170.9	222.4	94.8	141.4	173.5
274	34	0.05	5	0.0	0.0	0.2	0.0	0.0	0.2
			10	0.0	0.0	0.4	0.0	0.0	0.4
			20	0.2	7.1	46.3	0.2	4.4	46.8
550	60	0.05	5	0.0	0.3	2.1	0.0	0.3	2.0
			10	1.2	6.8	19.2	1.1	6.5	19.0
			20	28.3	83.9	163.9	28.9	79.9	123.1
880	113	0.06	5	0.0	0.0	0.4	0.0	0.0	0.4
			10	0.0	0.1	2.3	0.0	0.1	2.3
			20	0.1	5.5	37.0	0.2	5.2	38.3

Table S7.

Equilibrium swelling ratio of LBL hydrogels at 37°C. $Q_{eq} = W_{wet}/W_{dry}$

$\boldsymbol{n_{bb}}$	$\boldsymbol{n_L}$	$\boldsymbol{\phi_L}$	$\boldsymbol{Q_{eq}}$
274	79	0.12	15.0
550	60	0.05	23.9
	310	0.2	12.5
880	542	0.22	15.1

Table S8.

Mechanical properties of materials in Fig. 5C.

**Curve number**	**Definition**	$\boldsymbol{E\ (kPa)}$	$\boldsymbol{E_0\ (kPa)}$	$\boldsymbol{\beta}$	**Refs.**
**1**	Fetal Membrane	1.3	19.8	0.79	43
**2**	Chicken Gut	3.4	5.6	0.29	43
**3**	LBL hydrogel (DA)	39	26	0.16	This work
**4**	LBL hydrogel (UV)	5.1	5.8	0.18	This work
**5**	Linear hydrogel	28.8	36.1	0.0028	62
**6**	Linear hydrogel	2.9	3.3	0.0236	63

Fitting equation is given as

$$\sigma_{true} = \frac{1}{9}E(\lambda^2 - \lambda^{-1})\left[1 + 2\left(1 - \frac{\beta}{3}(\lambda^2 + 2\lambda^{-1})^{-2}\right)\right] \quad (8)$$

The equation provides relationship between the uniaxial true stress and polymer network elongation $\lambda = L/L_0$. The equation includes two fitting parameters that correspond to network structural modulus (E) and firmness (β). Both parameters define the so-called Young's modulus (the stress-strain slope at $\lambda \to 1$) as $E_0 = E(1 + 2(1 - \beta)^{-2})/3$.

Table S9.

GPC data of brush-on-brush (LBoBL) polymers.

**Entry**	$M_{n,\ GPC}$	M_w/M_n	Nomenclature
$PNiPAM_{1200}$-b-P(BiBEM-g-$OEO_3A_{13})_{144}$-co-OEO_3MA_{255})-b-$PNiPAM_{1200}$	333,000	2.01	LBoBL-399-13-31%
$PNiPAM_{1200}$-b-P(BiBEM-g-$OEO_3A_{24})_{144}$-co-OEO_3MA_{255})-b-$PNiPAM_{1200}$	657,000	2.17	LBoBL-399-24-22%
$PNiPAM_{1200}$-b-P(BiBEM-g-$OEO_3A_{67})_{144}$-co-OEO_3MA_{255})-b-$PNiPAM_{1200}$	623,500	2.08	LBoBL 399-67-10%
$PNiPAM_{1200}$-b-P(BiBEM-g-$OEO_3A_{126})_{144}$-co-OEO_3MA_{255})-b-$PNiPAM_{1200}$	734,000	2.16	LBoBL-399-126-6%

Movie S1.

Gelation time at $37°C$ for three backbones with degree of polymerization $n_{bb} \approx 274, 550, and\ 880$.

Movie S2.

Prompt and shape persistent gelation upon injection in $37\ °C$.
